# Regulation of P53 signaling in breast cancer by the E3 ubiquitin ligase RNF187

**DOI:** 10.1038/s41419-022-04604-3

**Published:** 2022-02-14

**Authors:** Xin Li, Zhiguo Niu, Chen Sun, Shu Zhuo, Huijie Yang, Xiao Yang, Yun Liu, Cheng Yan, Zhongbo Li, Qi Cao, Guimei Ji, Yinlu Ding, Ting Zhuang, Jian Zhu

**Affiliations:** 1 Xinxiang Key Laboratory of Tumor Migration and Invasion Precision Medicine, Xinxiang Medical University, Xinxiang, 453003 Henan Province P. R. China; 2 Henan Key Laboratory of Immunology and Targeted Therapy, School of Laboratory Medicine, Henan Collaborative Innovation Center of Molecular Diagnosis and Laboratory Medicine, School of Laboratory Medicine, Xinxiang Medical University, Xinxiang, 453003 Henan Province P. R. China; 3 Department of Neurosurgery, The First Affiliated Hospital of Zhengzhou University, Zhengzhou, Henan 450052 P. R. China; 4 Department of Developmental Biology, Harvard School of Dental Medicine, Harvard Stem Cell Institute, Boston, MA 02115 USA; 5 School of Medicine, Xinxiang University, Xinxiang, 453003 Henan P. R. China; 6 Centre for Transplant and Renal Research, Westmead Institute for Medical Research, The University of Sydney, Sydney, NSW Australia; 7 Department of General Surgery, The Second Hospital, Cheeloo College of Medicine, Jinan, Shandong Province P. R. China

**Keywords:** Breast cancer, Ubiquitylation

## Abstract

The tumor suppressor P53 plays critical role in preventing cancer. P53 is rarely mutated and remains functional in luminal-type breast cancer(1). According to current knowledge, wild-type P53 function is tightly controlled by posttranslational modifications, such as ubiquitination. Several ubiquitin ligases have been shown to regulate P53 ubiquitination and protein stability. Here, we report that RNF187, a RING family ubiquitin ligase, facilitates breast cancer growth and inhibits apoptosis by modulating P53 signaling. RNF187 expression was elevated in breast cancer and correlated with breast cancer survival only in the P53 wild-type groups. Bioinformatic analysis showed that the expression of RNF187 was negatively correlated with the expression of P53 target genes, such as IGFBP3 and FAS, in breast cancer. RNF187 depletion inhibited breast cancer growth and facilitated cell death. RNA sequencing analysis indicated that RNF187 could be an important modulator of P53 signaling. Further experiments showed that RNF187 interacts with P53 and promotes its degradation by facilitating its polyubiquitination in breast cancer cells. Interestingly, the in vitro ubiquitin assay showed that RNF187 can directly ubiquitinate P53 in a manner independent of MDM2. These findings reveal a novel direct P53 regulator and a potential therapeutic target for breast cancer.

## Introduction

Breast cancer is the most common cancer in women worldwide, while chemotherapy is one of the major treatment options for breast cancer patients, especially those with refractory cases [1]. P53 signaling was discovered more than 40 years ago and is always activated and subsequently involved in the cell cycle arrest and apoptosis caused by genotoxic drugs, such as cisplatin [2, 3]. Clinically, the P53 status is an important predictive marker of the chemotherapy response [4]. Mutant P53 can be used as a chemotherapy marker for a basal cell-like breast cancer [5]. Activation of P53 could be investigated as a therapeutic target. However, although many efforts have been made, targeting P53 signaling is still an immature strategy in breast tumors. Thus, it is necessary to further characterize P53 signaling in breast cancer to identify novel therapeutic strategies.

The P53 protein is composed of 393 amino acids and includes a transactivation domain, DNA binding domain, nuclear localization domain, and nuclear export domain [6]. When the P53 protein is activated by DNA damage or oxidative stress, its half-life increases, leading to apoptosis or accelerated cellular senescence [7], and enhanced expression of P53 target genes, such as P21, P53INP1, and FAS [8]. Subsequently, P53 activation causes either cell cycle arrest or apoptosis via [9, 10]. Besides, P53 activity can induce p21 dependent and independent growth arrest and cellular senescence [11]. In breast cancer, P53 is mutated in ~30% of tumors [12]. In patients receiving chemotherapy and those not receiving hormone therapy, wild-type TP53 status significantly decreased their overall survival [13]. Interestingly, P53 is more frequently mutated in ER alpha-negative subtypes than in ER alpha-positive subtypes (≈50% vs ≈15%) [14, 15]. Most ER-positive breast tumors harbor wild-type P53, which can function to regulate ER alpha gene expression [16].

The P53 protein is under precise control with a half-life of ~20 min [17]. Several ubiquitin ligases, including MDM2, COP1, and CHIP, have been shown to directly modulate P53 protein stability [18–20]. The most well-known is MDM2 [18]. Once P53 is activated, it induces the expression of MDM2, which subsequently interacts with P53 and facilitates its polyubiquitination and degradation [21]. The MDM2-P53 loop effectively maintains homeostasis and proper response to certain stimuli. In addition, quite a few E3 ligases, such as SMURF1 and RBCK1, have been found to modify the MDM2-P53 complex and indirectly regulate P53 stability [22, 23]. Our previous study showed that the RNF31 and SHARPIN proteins promote P53 degradation by regulating MDM2 stability [24, 25].

RNF187 (RING finger 187), which is also called RING domain AP1 coactivator-1, is a RING family ubiquitin ligase [26]. Our previous study showed that RNF187 functioned as a negative regulator in triple-negative breast cancer by modulating Hippo signaling [27]. However, we observed the opposite cellular phenotype in ER alpha-positive breast cancer cells. Unbiased analysis of global gene expression data showed that RNF187 is a novel regulator of the P53 protein. RNF187 directly promotes P53 polyubiquitination and degradation, leading to suppression of P53 signaling and finally facilitating breast cancer cell growth and inhibition of cisplatin-induced apoptosis.

## Results

### RNF187 is elevated in breast tumors and is required for cancer growth and anti-apoptosis in luminal-type breast cancer

**Fig. 1:**
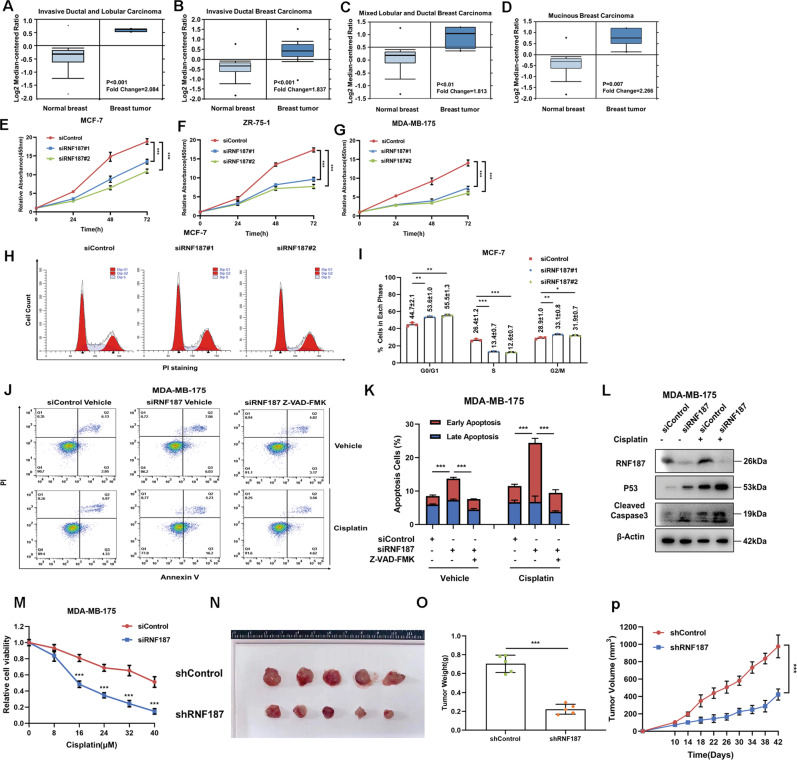
RNF187 is elevated in breast tumors and is required for cancer cell growth and apoptosis resistance in luminal-type breast cancer. **A**–**D** RNF187 mRNA levels are elevated in breast cancer samples compared with normal breast tissues in the ONCOMINE database (http://oncomine.org). **E** RNF187 depletion inhibits the proliferation of breast cancer cells. MCF-7 cells were transfected with 50 nM RNF187 siRNA or 50 nM control siRNA. Two independent siRNAs were used. After 24 h, a CCK-8 assay was used to determine the cellular metabolic activity at the indicated time points after transfection. Experiments were performed in triplicate. *P < 0.05; **P < 0.01; ***P < 0.001 for cell growth comparisons. **F** RNF187 depletion inhibits the proliferation of breast cancer cells. MDA-MB-175 cells were transfected with 50 nM RNF187 siRNA or 50 nM control siRNA. Two independent siRNAs were used. After 24 h, a CCK-8 assay was used to determine the cellular metabolic activity at the indicated time points after transfection. Experiments were performed in triplicate. *P < 0.05; **P < 0.01; ***P < 0.001 for cell growth comparisons. **G** RNF187 depletion inhibits the proliferation of breast cancer cells. ZR751 cells were transfected with 50 nM RNF187 siRNA or 50 nM control siRNA. Two independent siRNAs were used. After 24 h, a CCK-8 assay was used to determine the cellular metabolic activity at the indicated time points after transfection. Experiments were performed in triplicate. *P < 0.05; **P < 0.01; ***P < 0.001 for cell growth comparisons. **H**, **I** Cell cycle analysis to assess the effect of RNF187 knockdown in MCF-7 cells. MCF-7 cells were transfected with 50 nM RNF187 siRNA or 50 nM control siRNA. Two independent siRNAs were used. After 24 h, the cells were harvested, fixed with 70% ethanol, and stained with propidium iodide. The cells were subjected to FACS analysis. Experiments were performed in triplicate. *P < 0.05; **P < 0.01; ***P < 0.001 for cell proportion comparisons. Representative histograms and cell cycle phase distributions are shown in Fig. 1I, J, respectively. **J**, **K** RNF187 depletion promoted apoptosis in MDA-MB-175 cells, which effect could be rescued by the caspase-3 inhibitor (Z-VAD-FMK). MDA-MB-175 cells were transfected with siControl or siRNF187. After that, cells were treated with vehicle or 10 uM cisplatin in combination with 20 uM Z-VAD-FMK. After 24 h, cells were stained with PI and Annexin V. Then cells were subject to FACS analysis for the proportion of apoptotic cells. Each group was done in triplicates. *P < 0.05; **P < 0.01; ***P < 0.001 for comparison. **L** RNF187 depletion increased the cleaved caspase-3 protein level under both vehicle and cisplatin treatment conditions. MDA-MB-175 cells were transfected with siControl or siRNF187. Cisplatin (10 μM) was added to treat the cells for 6 h. Then, the cells were harvested for western blot analysis. RNF187, P53, and cleaved caspase-3 protein levels were determined by western blot analysis. Actin was used as the internal control. **M** RNF187 depletion sensitizes MDA-MB-175 cells to cisplatin-mediated inhibition. MDA-MB-175 cells were transfected with 50 nM siRNF187 or siControl. After 24 h, the cells were treated with cisplatin at the indicated concentration for 24 h. Cell viability was determined via a CCK-8 assay. **N**–**P** RNF187 depletion inhibits breast tumor growth in vivo. MCF-7 cells were stably transduced with lentiviral vectors carrying scrambled shRNA or RNF187 shRNA. Female NOD scid gamma (NSG) mice were implanted with slow-release 17β-estradiol pellets (0.72 mg/90-d release; Innovative Research of America) 1 day before MCF-7 tumor cell injection into the mammary fat pad (2 × 10^6^ MCF-7 cells suspended in 100 µl of Matrigel solution). MCF-7 tumor xenografts were measured every 3–4 days, and tumor volumes were calculated as length × width^2^/2. Mice were sacrificed 6 weeks after tumor cell injection. Tumor growth curves, weights, and photographs are shown in panels **N**–**P**, respectively.

We first analyzed RNF187 expression in breast cancer. Analysis of the Oncomine database showed that RNF187 was elevated in breast tumors compared with normal breast tissues (Fig. 1A–D, https://www.oncomine.org). Since our previous study showed that RNF187 played tumor suppressor roles in triple-negative breast cancer, we further investigated its role in luminal-type breast cancer [27]. MCF-7, MDA-MB-175, and ZR751 cells, all of which are ER alpha-positive and P53 wild type [28], were utilized as the models in this study [28]. The CCK-8 assay showed that RNF187 depletion significantly inhibited the growth of MCF-7, MDA-MB-175, and ZR751 cells (Fig. 1E–G). Cell cycle analysis showed that RNF187 depletion via two independent siRNAs caused G1 arrest (Fig. 1H–I). Since MCF-7 cells are deficient in caspase-3, which is a critical factor in apoptosis, we carried out a cell death assay in MDA-MB-175 cells. Annexin V/PI double staining coupled with FACS analysis indicated that RNF187 depletion increased the proportion of apoptotic cells, which effect could be further enhanced by cisplatin treatment (Supplementary Fig. 2A, B). Interestingly, when we utilized the caspase-3 inhibitor Z-VAD-FMK to treat the MDA-MB-175 cells, the caspase-3 inhibitor could bring back the apoptotic cell numbers caused by RNF187 depletion (Fig. 1J, K). The beta-Gal assay showed that RNF187 depletion could significantly enhance breast cancer cell senescence (Supplementary Fig. 1A, B). Immunoblotting showed that RNF187 depletion increased the cleaved caspase-3 level in MDA-MB-175 cells. Besides, RNF187 depletion also increased P53 levels in both vehicle and cisplatin-treated conditions (Fig. 1L). The immunostaining showed that RNF187 depletion significantly increased the number of caspase-3 positive cells (Supplementary Fig. 1C, D). In addition, RNF187 depletion significantly sensitized MDA-MB-175 cells to cisplatin-induced cell death (Fig. 1M). Then, we generated a model of stable RNF187 silencing in MCF-7 cells and further investigated the role of RNF187 in vivo by establishing a xenograft mouse model. Our data showed that RNF187 depletion reduced the tumor growth rate in vivo (Fig. 1N–P).

### Bioinformatic analysis reveals the correlation between RNF187 and P53 signaling in breast cancer cells

**Fig. 2:**
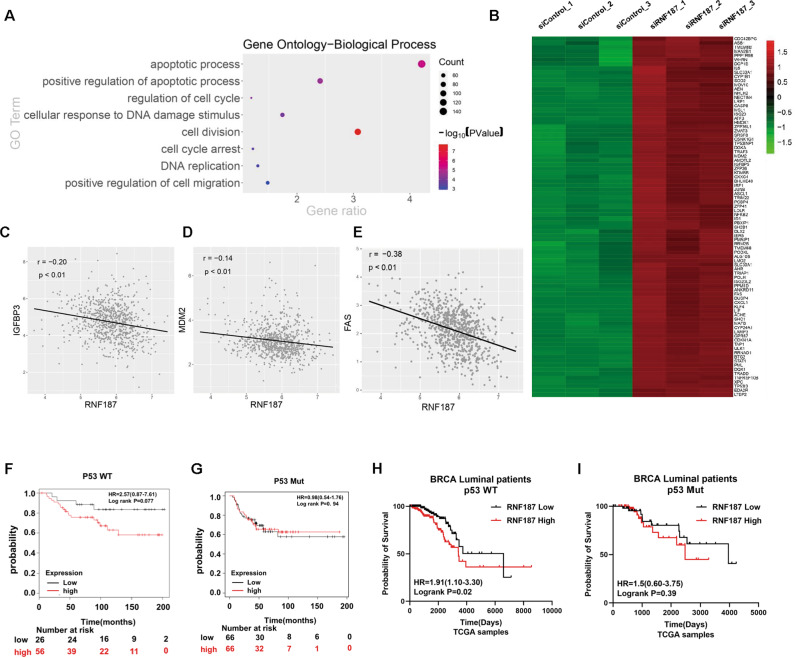
Bioinformatic analysis reveals the correlation between RNF187 and P53 signaling in breast cancer cells. **A** Gene ontology analysis of the RNA sequencing data shows that RNF187 depletion in breast cancer cells activates the apoptotic process and cell cycle arrest. MCF-7 cells were transfected with siRNF187 or siControl. After 48 h, total mRNA was extracted for RNA sequencing analysis. The siControl and siRNF187 groups were analyzed in triplicate. **B** The heat map shows the P53 target genes, whose expression is significantly increased by RNF187 depletion in MCF-7 cells. **C**–**E** Publicly available data show that RNF187 expression is inversely correlated with that of the p53 target genes IGFBP3, MDM2, and FAS (https://tcga-data.nci.nih.gov/tcga/). **F**, **G** The Kaplan–Meier curve of progression-free survival shows that RNF187 tends to be related to poor prognosis in breast cancer patients harboring wild-type P53, but no correlation was found in the P53 mutant groups. The data were generated from the KMPLOT database (https://kmplot.com). **H**, **I** The Kaplan–Meier curve of overall survival shows that RNF187 correlates with poor survival in a luminal-type of breast cancer patients harboring wild-type P53, but no correlation was found in the P53 mutant groups of luminal-type breast cancers. The data were generated from the TCGA database (https://tcga-data.nci.nih.gov/tcga/).

Since we showed that RNF187 is an important factor in maintaining cell growth and survival in luminal-type cancers, we further investigated the function of RNF187 via an unbiased approach. We depleted RNF187 in MCF-7 cells for whole-genome expression analysis. Gene ontology-biological process analysis showed that RNF187 depletion affects several aspects of cancer biological processes, including apoptosis, cell cycle arrest, and the DNA damage response (Fig. 2A). Since P53 has been shown to be important in all these aspects, we further investigated the changes in the expression of P53 target genes at the whole-genome level. Interestingly, the heat map showed that RNF187 depletion significantly activated a large group of P53 target genes, including CDKN1A, MDM2, and FAS (Fig. 2B). Based on the potential link between RNF187 and P53, we further investigated the relevance of the clinical data. In the TCGA database, we observed that the expression of RNF187 was inversely correlated with the expression of several P53 target genes, such as MDM2, FAS, and IGFBP3 (Fig. 2C–E). Finally, the prognostic data showed that RNF187 tended to relate to poor prognosis specifically in patients with P53 WT breast cancer (*P* = 0.07). However, there is no significant correlation between RNF187 and prognosis in the P53 mutant groups (*P* = 0.94; Fig. 2F, G). We further analyzed the prognosis in ER-positive breast cancer patients. The TCGA data showed that RNF187 correlated with poor survival in P53 WT/Luminal-type patients (*P* = 0.02), but no correlation in P53 mutant/luminal-type patients (*P* = 0.39; Fig. 2H, I). All these data indicate that RNF187 could be an important factor in P53 function in breast cancer.

In order to confirm the functional correlation of RNF187 and P53 status, we investigated the effect of RNF187 in the luminal-type of breast cancer cells with P53 mutation. Thus, we utilized T47D cells (ER-positive and P53 mutant) to carry out further experiments. The data showed that RNF187 depletion could be achieved in T47D cells, but no dramatic change in P53 protein level in T47D cells (Supplementary Fig. 3A, B). Besides, RNF187 depletion could not affect cell proliferation in T47D cells (Supplementary Fig. 3C). The cell cycle analysis showed that RNF187 silencing could not affect the cell cycle progression in T47D cells (Supplementary Fig. 3D, E). The PI/Annexin V double staining coupled with FACS analysis indicated that RNF187 could not increase apoptotic cell proportion in T47D cells (Supplementary Fig. 3F, G). Based on these data, we can conclude that P53 status is important for RNF187 to exert its effects on cell proliferation and apoptosis in Luminal-type of breast cancers.

### RNF187 depletion increases the P53 protein level and P53 target gene expression in breast cancer cells

**Fig. 3:**
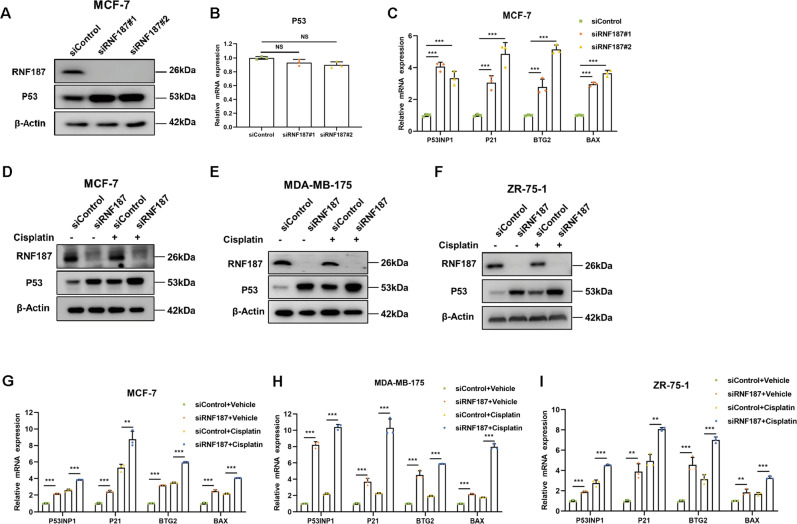
RNF187 depletion increases the P53 protein level and P53 target gene expression in breast cancer cells. **A**, **B** RNF187 depletion increases the P53 protein level but not the mRNA level in MCF-7 cells. MCF-7 cells were transfected with siControl or siRNF187. After 48 h, the cells were harvested for western blot analysis. RNF187 and P53 protein levels were determined by western blot analysis. Actin was used as the internal control. The P53 mRNA level was determined by qPCR, while 36B4 was used as the internal control. Each group was analyzed in triplicate. *P < 0.05; **P < 0.01; ***P < 0.001 for gene expression comparisons. **C** RNF187 depletion increases P53 target gene expression. MCF-7 cells were transfected with siControl or siRNF187. After 48 h, total RNA was extracted for gene expression analysis. Each group was analyzed in triplicate. *P < 0.05; **P < 0.01; ***P < 0.001 for target gene expression comparison. **D** RNF187 depletion increases the P53 protein level in MCF-7 cells under both vehicle and cisplatin treatment conditions. MCF-7 cells were transfected with siControl or siRNF187. After 48 h, the cells were treated with 10 μM cisplatin or vehicle for 6 h. Then, the cells were harvested for western blot analysis. RNF187 and P53 protein levels were determined by western blot analysis. Actin was used as the internal control. **E** RNF187 depletion increases the P53 protein level in MDA-MB-175 cells under both vehicle and cisplatin treatment conditions. MDA-MB-175 cells were transfected with siControl or siRNF187. After 48 h, the cells were treated with 10 μM cisplatin or vehicle for 6 h. Then, the cells were harvested for western blot analysis. RNF187 and P53 protein levels were determined by western blot analysis. Actin was used as the internal control. **F** RNF187 depletion increases the P53 protein level in ZR751 cells under both vehicle and cisplatin treatment conditions. ZR751 cells were transfected with siControl or siRNF187. After 48 h, the cells were treated with 10 μM cisplatin or vehicle for 6 h. Then, the cells were harvested for western blot analysis. RNF187 and P53 protein levels were determined by western blot analysis. Actin was used as the internal control. **G** RNF187 depletion increases P53 target gene expression under both vehicle and cisplatin treatment conditions. MCF-7 cells were transfected with siControl or siRNF187. After 48 h, 10 μM cisplatin was added to treat the cells for 6 h. Total RNA was extracted for gene expression analysis. Each group was analyzed in triplicate. *P < 0.05; **P < 0.01; ***P < 0.001 for target gene expression comparison. **H** RNF187 depletion increased P53 target gene expression under both vehicle and cisplatin treatment conditions. MDA-MB-175 cells were transfected with siControl or siRNF187. After 48 h, 10 µM cisplatin was added to treat the cells for 6 h. Total RNA was extracted for gene expression analysis. Each group was analyzed in triplicate. *P < 0.05; **P < 0.01; ***P < 0.001 for target gene expression comparison. **I** RNF187 depletion increased P53 target gene expression under both vehicle and cisplatin treatment conditions. ZR751 cells were transfected with siControl or siRNF187. After 48 h, 10 μM cisplatin was added to treat the cells for 6 h. Total RNA was extracted for gene expression analysis. Each group was analyzed in triplicate. *P < 0.05; **P < 0.01; ***P < 0.001 for target gene expression comparison.

We depleted RNF187 via two independent siRNAs in MCF-7 cells. The immunoblotting results showed that RNF187 depletion increased the P53 protein level in MCF-7 cells but did not significantly change the P53 mRNA level (Fig. 3A, B). Consistent with this finding, the qPCR results showed that RNF187 depletion increased the expression of classical P53 target genes, including P53INP1, BTG2, P21, and BAX (Fig. 3C). To test the effect of RNF187 under both normal and P53-activated conditions, we utilized cisplatin, a chemotherapeutic drug, to activate the P53 pathway in breast cancer cells. The immunoblotting results showed that RNF187 increased the P53 protein level in MCF-7, MDA-MB-175, and ZR751 cells treated with either vehicle or cisplatin (Fig. 3D–F). In addition, the qPCR results indicated that RNF187 depletion increased P53 target gene expression in both vehicle- and cisplatin-treated cells of all three cell lines (Fig. 3G–I).

### RNF187 modulates cell growth and apoptosis via P53 signaling

**Fig. 4:**
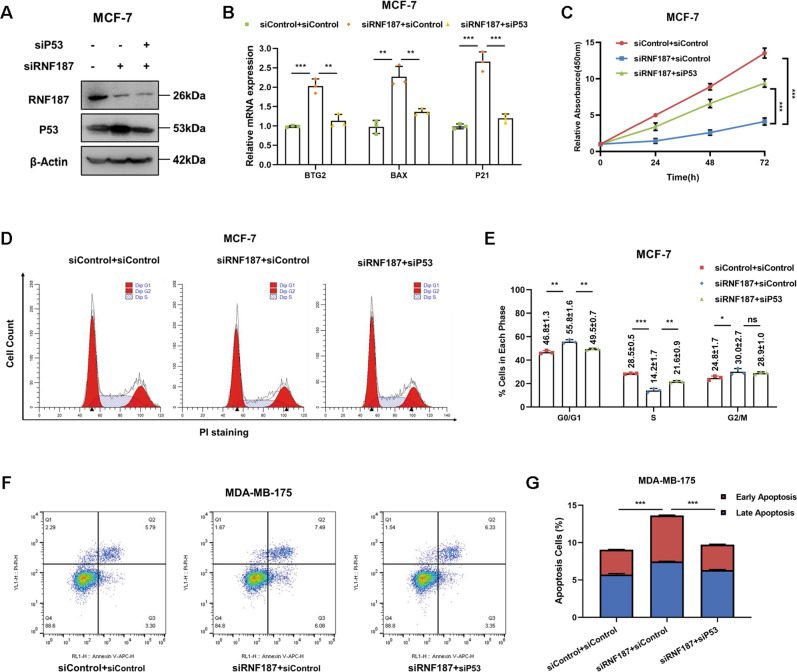
RNF187 modulates cell growth and apoptosis via P53 signaling. **A** RNF187 and P53 depletion effect in MCF-7 cells. MCF-7 cells were transfected with siControl, siRNF187, or siRNF187+siP53. After 48 h, the cells were harvested for western blot analysis. RNF187 and P53 protein levels were determined by western blot analysis. Actin was used as the internal control. **B** P53 depletion rescued the expression of its target genes inhibited by RNF187 knockdown. MCF-7 cells were transfected with siControl, siRNF187, or siRNF187+siP53. After 48 h, total RNA was extracted for gene expression analysis. Each group was analyzed in triplicate. *P < 0.05; **P < 0.01; ***P < 0.001 for target gene expression comparison. **C** Cell growth inhibition induced by RNF187 silencing was partially rescued by P53 depletion in MCF-7 cells. MCF-7 cells were transfected with 50 nM RNF187 siRNA, 50 nM control siRNA, or 50 nM siRNF187+siP53. After 24 h, a CCK-8 assay was used to determine the cellular metabolic activity at the indicated time points after transfection. Experiments were performed in triplicate. *P < 0.05; **P < 0.01; ***P < 0.001 for cell growth comparisons. **D**, **E** Cell cycle arrest caused by RNF187 silencing was partially rescued by P53 depletion in MCF-7 cells. MCF-7 cells were transfected with 50 nM RNF187 siRNA, 50 nM control siRNA, or 50 nM siRNF187+siP53. After 24 h, cells were harvested, fixed with 70% ethanol, and stained with propidium iodide. The cells were subjected to FACS analysis. Experiments were performed in triplicate. *P < 0.05; **P < 0.01; ***P < 0.001 for cell proportion comparisons. Representative histograms and cell cycle phase distributions are shown in Fig. 4D, E, respectively. **F**, **G** RNF187 depletion promoted apoptosis, and these effects were partially rescued by P53 depletion in MDA-MB-175 cells. MDA-MB-175 cells were transfected with 50 nM RNF187 siRNA, 50 nM control siRNA, or 50 nM siRNF187+siP53. After 24 h, cells were stained with PI and Annexin V. Then, cells were subjected to FACS analysis to determine the proportion of apoptotic cells. Each group was analyzed in triplicate. *P < 0.05; **P < 0.01; ***P < 0.001 for comparisons.

To investigate the logical link between the cancer phenotype and P53 signaling in terms of the function of RNF187, we carried out several rescue experiments. We simultaneously silenced P53 expression and depleted RNF187 and validated the P53 protein level and the expression of its target genes in MCF-7 cells (Fig. 4A, B). In Fig. 4A, it showed that RNF187 depletion could increase the P53 protein level, which could be rescued by further P53 knocking-down (Fig. 4A). The qPCR results showed that RNF187 depletion increased the expression of classical P53 target genes, which effect could be brought back via further P53 silencing (Fig. 4B). The data in Fig. 4C show that RNF187 knockdown in MCF-7 cells inhibited breast cancer growth and that the effect was at least partially rescued by further P53 silencing. The cell cycle analysis results showed that the G1 arrest caused by RNF187 depletion was at least partially alleviated by the P53 knockdown (Fig. 4D, E). In addition, the results of annexin V/PI staining coupled with FACS analysis showed that P53 silencing at least partially rescued the apoptosis caused by RNF187 depletion in MDA-MB-175 cells (Fig. 4F, G).

### RNF187 associates with P53 independent of MDM2

**Fig. 5:**
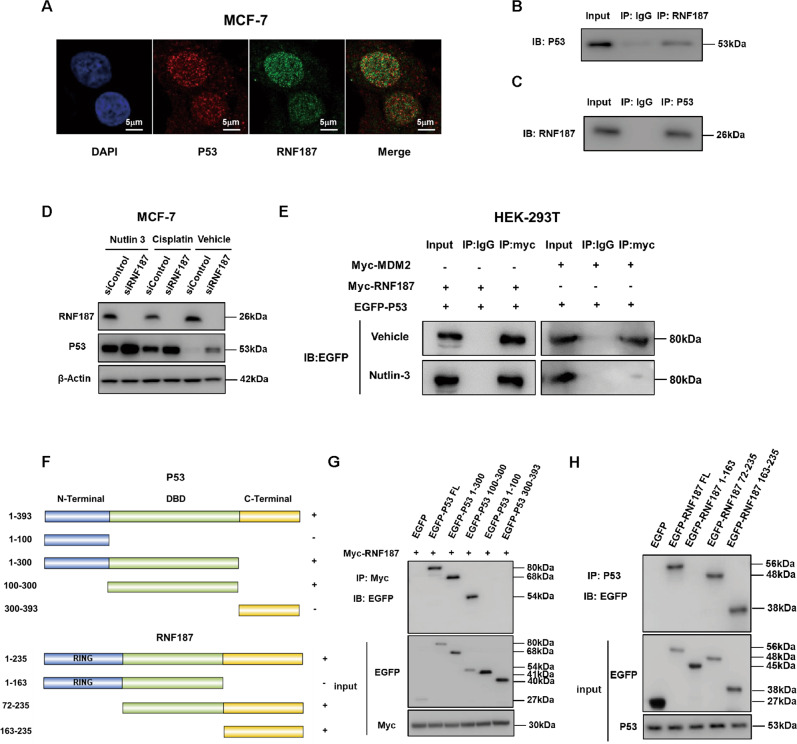
RNF187 associates with P53 independent of MDM2. **A** Intracellular localization analysis of P53 and RNF187 by immunofluorescence assay. MCF-7 cells were cultured in a normal medium before fixation. Intracellular localization of RNF187 (green) and P53 (red) is shown. Nuclei (blue) were stained with 4′,6-diamidino-2-phenylindole (DAPI). **B**, **C** Co-IP assay revealed an association between endogenous RNF187 and P53 in MCF-7 cells. MCF-7 cells were harvested with RIPA lysis buffer. Co-IP was performed using antibodies as indicated. **D** The difference in the P53 protein level between siControl and RNF187 cells could not be diminished by Nutlin-3. MCF-7 cells were transfected with 50 nM RNF187 siRNA and 50 nM control siRNA. After 24 h, the cells were treated with 10 μM Nutlin-3 or 10 μM cisplatin for 2 h. Then, the cells were harvested for western blot analysis. RNF187 and P53 protein levels were determined by western blot analysis. Actin was used as the internal control. **E** Nutlin-3 could not block the interaction between RNF187 and P53. HEK293 cells were transfected with Myc-RNF187, Myc-MDM2, and EGFP-P53. After 48 h, the cells were treated with 10 μM Nutlin-3 for 2 h. Then, HEK293 cells were harvested with RIPA lysis buffer. Co-IP was performed using the indicated antibody. **F** P53 domain structure and deletion mutants were used in this study, and RNF187 full-length and deletion mutants were used in this study. **G** P53 interacts with RNF187 through its DNA binding domain. HEK293 cells were transfected with 2 µg of Myc-RNF187 together with full-length or mutants (ΔN-terminal, ΔN-terminal+ΔDBD, ΔC-terminal, and ΔC-terminal+ΔDBD). After 24 h, the cells were treated with 10 μM MG132 for 6 h. Then, the cells were harvested with NP-40 lysis buffer. Co-IP was performed using Myc antibody. The possible interacting P53 domains were detected with an anti-GFP antibody. **H** The C-terminus (163–235) is required for RNF187 to interact with P53. HEK293 cells were transfected with 2 µg of P53 together with full-length GFP-RNF187 or mutant GFP-RNF187 (1-235, 1-163, 72-235, 163–235). After 24 h, the cells were treated with 10 μM MG132 for 6 h. Then, the cells were harvested with NP-40 lysis buffer. Co-IP was performed using an anti-P53 antibody. The possible interacting RNF187 domains were detected with an anti-GFP antibody.

We further investigated the localization of RNF187 and P53 in breast cancer cells. Immunostaining showed that both RNF187 and P53 were localized mainly in the nucleus (Fig. 5A). Endogenous immunoprecipitation showed that RNF187 interacted with P53 in MCF-7 cells (Fig. 5B, C). Since several studies have shown that quite a few E3 ligases modulate P53 function via the MDM2 protein, we sought to clarify whether MDM2 is involved in the regulatory relationship between P53 and RNF187. We utilized nutlin-3, which is an effective blocker of the P53–MDM2 interaction. Interestingly, nutlin-3 could not diminish the difference in the P53 protein level between siRNF187 and siControl cells, unlike the observations in our previous RNF31-P53 study (Fig. 5D). In contrast with its effect on blocking the P53–MDM2 interaction, nutlin-3 treatment had little effect on blocking the P53–RNF187 interaction (Fig. 5E). These data showed that RNF187 modulates the P53 protein level independent of MDM2. We further characterized the interaction domains between RNF187 and P53. RNF187 is composed of two functional domains, specifically, a RING domain (aa 1–72) and a protein interaction domain (aa 163–235), while P53 contains three functional domains: a transactivation domain at the N-terminus (aa 1–100), a DNA binding domain (aa 100–300), and a nuclear localization/export domain (aa 300–393). We constructed P53 mutants and RNF187 mutants (Fig. 5F). The immunoprecipitation results implied that the DNA binding domain of P53 is required for its interaction with RNF187 (Fig. 5G), while the C-terminal domain of RNF187 is required for its interaction with P53 (Fig. 5H).

### RNF187 promotes P53 K48-linked polyubiquitination and degradation

**Fig. 6:**
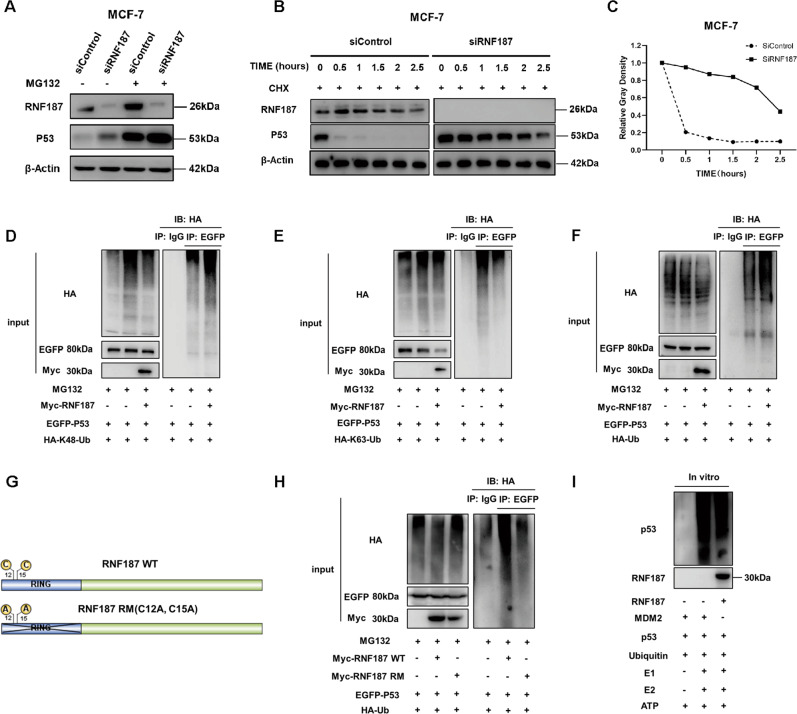
RNF187 promotes P53 K48-linked polyubiquitination and degradation. **A** In the presence of the proteasome inhibitor MG132, RNF187 does not further increase the P53 protein level. MCF-7 cells were transfected with 50 μM siControl or siRNF187. After 24 h, the cells were treated with 10 μM MG132/vehicle for 6 h. Cell lysates were prepared for western blot analysis. The results are representative of three independent experiments. **B**, **C** RNF187 depletion increases the P53 half-life in MCF-7 cells. MCF-7 cells were transfected with 50 nM siControl or siRNF187. After 24 h, cells were treated with 100 µM cycloheximide/vehicle for the indicated times. Cell lysates were prepared for western blot analysis. The results are representative of three independent experiments. The relative P53 density was measured by ImageJ software. **D** RNF187 increases the K48-linked polyubiquitination of P53. HEK293 cells were transfected with 2 µg of P53 plasmid, 0.5 µg of HA-K48 Ubi plasmid, and 0.5 µg of Myc-tag or Myc-RNF187 plasmids. The cell extracts were immunoprecipitated with an anti-HA antibody. K48-specific polyubiquitinated P53 was detected via western blot analysis. **E** RNF187 decreases K63-linked polyubiquitination of P53. HEK293 cells were transfected with 2 µg of P53 plasmid, 0.5 µg of HA-K63 Ubi plasmid, and 0.5 µg of Myc-tag or Myc-RNF187 plasmids. The cell extracts were immunoprecipitated with an anti-HA antibody. K63-specific polyubiquitinated P53 was detected via western blot analysis. **F** RNF187 increases the total polyubiquitination of P53. HEK293 cells were transfected with 2 µg of P53 plasmid, 0.5 µg of HA-Ub plasmid, and 0.5 µg of Myc-tag or Myc-RNF187 plasmids. The cell extracts were immunoprecipitated with an anti-HA antibody. Total polyubiquitinated P53 was detected via western blot analysis. **G** The wild-type and mutant forms of RNF187 were used in the study. The mutation sites (C12A; C15A) in the RING domain diminished the ubiquitin ligase function of RNF187. **H** The ubiquitin ligase functional RING domain is required for RNF187 to promote P53 polyubiquitination. RNF187 increases the total polyubiquitination of P53. HEK293 cells were transfected with 2 µg of P53 plasmid, 0.5 µg of HA-Ub plasmid, and 0.5 µg of Myc-tag or Myc-RNF187 WT or RING mutant form plasmids. The cell extracts were immunoprecipitated with an anti-HA antibody. Total polyubiquitinated P53 was detected via western blot analysis. **I** The in vitro ubiquitination assay showed that RNF187 can directly promote P53 polyubiquitination. Ubiquitination was analyzed with a ubiquitination kit (Boston Biochem). The recombinant proteins were mixed with E1, ATP, ubiquitin solution, and E2 enzyme in a final volume of 20 µl reaction buffer. MDM2 was used as the positive control. Ubiquitination of P53 was analyzed with an anti-P53 antibody.

Since RNF187 was shown to interact with P53 in breast cancer cells, we further investigated the potential molecular mechanisms. RNF187 depletion increased the P53 protein level, and this effect was diminished by treatment with the proteasome inhibitor MG132 (Fig. 6A). This result might indicate that RNF187 modulates the level of the P53 protein via its stability. Then, we utilized cycloheximide, a protein synthesis inhibitor, to assess protein stability. The data in Fig. 6B show that RNF187 depletion significantly increased the half-life of endogenous P53 (Fig. 6B, C). According to the isopeptide binding to the lysines of ubiquitin, the ubiquitination manners include K9, K11, K27, K29, K33, K48, K63, and linear ubiquitinations. In general, the K48-linked ubiquitination is the classical pathway for protein degradation, while the K63-linked ubiquitination could modulate several cellular processes, such as protein trafficking and DNA damage response. P53 is predominantly ubiquitinated in a K48-linked manner for degradation, while K63-linked ubiquitination of P53 enhances its stability and inhibits K48-linked ubiquitination. We further investigated the effect of RNF187 on P53 ubiquitination. We overexpressed P53, ubiquitin plasmid, and RNF187/control plasmids in HEK293 cells. We immunoprecipitated P53 and detected the P53 polyubiquitination against ubiquitin. The ubiquitin-based immunoprecipitation assay showed that RNF187 facilitated total and K48-linked polyubiquitination of P53 but inhibited K63-linked polyubiquitination of P53 (Fig. 6D–F). Furthermore, we constructed the E3 ligase dominant-negative mutant of RNF187 (C12A; C15A) and carried out a ubiquitin-based immunoprecipitation assay (Fig. 6G). The data showed that the ubiquitin ligase activity of RNF187 is required for inducing P53 polyubiquitination (Fig. 6H). To show whether RNF187 is a direct E3 ligase for P53 polyubiquitination, an in vitro ubiquitination assay was carried out with purified substrates and E3 ligases, while MDM2 was utilized as the positive control. The data showed that RNF187 directly promoted P53 polyubiquitination in vitro (Fig. 6I).

## Discussion

**Fig. 7:**
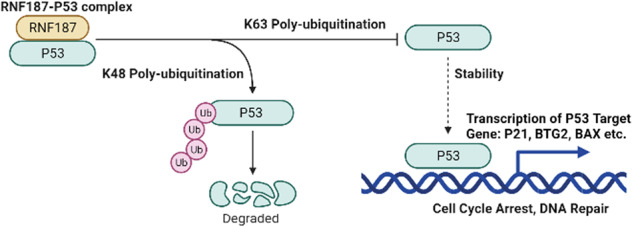
The hypothetical model of the mechanism by which RNF187 regulates P53 signaling in breast carcinoma. RNF187 interacts with the P53 protein and directly promotes its K48-linked polyubiquitination and degradation, which subsequently inhibits P53 target gene expression and facilitates breast cancer progression.

In our current study, we report that the RING family E3 ubiquitin ligase RNF187 acts as an endogenous inhibitor of P53 signaling in breast cancer. RNF187 expression was elevated in breast cancer samples, related to poor prognosis in P53 WT breast cancer patients, and correlated with P53 target gene expression in human breast cancer samples. RNF187 facilitated breast cancer cell growth and apoptosis resistance in both cell culture and xenograft mouse models. The molecular biological studies showed that RNF187 directly promoted the K48-linked polyubiquitination and proteasome-dependent degradation of P53 in breast cancer (Fig. 7). In summary, we propose that RNF187 inhibition, which subsequently facilitates P53 signaling and induces cell cycle arrest and apoptosis, could be a therapeutic target for patients with P53 wild-type breast cancer.

The TP53 gene was identified more than 40 years ago. As the guardian of the genome, P53 protects cells against malignant transformation by inducing cell cycle arrest and DNA repair processes or apoptosis [29]. P53 mutations, which exist in 2–90% of different human cancers, always result in loss of its tumor suppressor function and gain of an oncogenic function in human cancers [30, 31]. Several studies have shown that mutant P53 proteins become more stable and accumulate in the nucleus, which disrupts the functions of DNA repair complexes and certain tumor suppressors, such as P63 and P73 [32, 33]. Interestingly, the mutation frequency of P53 shows a dramatic difference across the subtypes of breast tumors. For example, P53 is mutated in 15% of ER alpha-positive breast tumors but 80% of triple-negative breast cancers [12, 14]. This difference might indicate that P53 is mostly functional in luminal-type human breast cancers and that activation of P53 signaling could be a promising therapeutic strategy for luminal-type breast cancer.

The wild-type P53 protein is subject to several kinds of posttranslational modifications, such as ubiquitination, acetylation, and phosphorylation [34, 35]. Among these modifications, ubiquitination, which is tightly linked to protein stability and degradation, is attracting attention in the cancer research field. K48-linked ubiquitination, the major ubiquitination modification, always leads to protein degradation, while some other types of ubiquitination modifications, such as K63-linked ubiquitination and monoubiquitination, are reported to modulate P53 function [36, 37]. A group of E3 ligases, including MDM2 and COP1, have been reported to directly ubiquitinate the P53 protein [20]. The most frequently reported example is the MDM2-P53 complex, in which MDM2 interacts with P53 and promotes P53 ubiquitination at several lysine sites located in the DNA binding domain and C-terminus, while P53 can bind to the promoter region of the MDM2 gene and facilitate MDM2 protein expression [38]. However, recent studies have reported that a large group of E3 ubiquitin ligases, including RNF2, RNF31, and SHARPIN, promote P53 ubiquitination and degradation by enhancing MDM2 function [24, 25, 39].

The E3 ubiquitin ligase RNF187 has been reported to regulate RAS-AP1 signaling and facilitate cancer progression [40]. In addition, RNF187 can also function as a coactivator for AP1 activation and promote cell cycle progression by inhibiting K48-linked polyubiquitination of CDC2 and cyclin D1 [26]. Our research focused on RNF187 function in human breast cancer, and we discovered that RNF187 functions as an inhibitor of the Hippo/YAP axis, which subsequently inhibits cancer progression in triple-negative breast cancers [27]. However, we found an opposite cancer phenotype in luminal-type cancer models, in which RNF187 was required for cell growth and apoptosis resistance. Interestingly, RNF187 directly polyubiquitinated the P53 protein for degradation, and this effect was independent of MDM2 function. Our findings revealed not only a novel E3 ligase regulator of P53 signaling but also the “multifaceted” nature of RNF187 in different subtypes of breast cancer cells.

In conclusion, we identified an interesting E3 ligase, RNF187, that facilitates wild-type P53 degradation in breast cancer cells. RNF187 can promote breast cancer cell invasion and proliferation by promoting P53 degradation. As a novel modulator of P53 signaling, disrupting RNF187 activity or affecting RNF187 expression could be a plausible approach to treat luminal-type breast cancer.

## Materials and methods

### Cell culture

MCF-7, MDA-MB-175, ZR751, and HEK293 cells were acquired from the American Type Culture Collection (ATCC). ZR751 cells were cultured in RPMI-1640 medium (42401, Life Technologies) supplemented with 2 mM l-glutamine (25030, Life Technologies) and 10% FBS. MDA-MB-175, HEK293, and MCF-7 cells were cultured in Dulbecco’s modified Eagle’s medium containing 4.5 g/L glucose and 4 mM l-glutamine (DMEM, 41965, Life Technologies) and supplemented with 10% fetal bovine serum (FBS, 10270, Life Technologies). All cell lines were characterized by cell line authentication. Cell line authentication was performed via short tandem repeat (STR) profiling in a PowerPlex 21 system. The STR data of the MCF-7, MDA-MB-175, ZR751, and HEK293 cell lines were consistent with the STR data from ATCC.

### RNA isolation and quantitative real-time PCR (qRT-PCR)

Total RNA was extracted with an RNeasy Plus Mini Kit (Qiagen, China; Cat: 4992235) following the manufacturer’s specifications. Reverse transcription was performed using a RevertAid First Strand cDNA Synthesis Kit (Thermo, Lithuania). qRT-PCR was carried out using GoTaq^®^ qPCR Master Mix (Promega, USA) in a 7500 Fast Real-Time PCR System (Applied Biosystems, Singapore). The 36B4 gene was used as the internal control. The sequences of the primers used for qPCR are listed in Supplementary Table 1.

### Western blot analysis

Standard western blotting procedures were used to analyze protein expression in cells. The following antibodies were used for western blot analysis: anti-GFP (AB290, Abcam, 1:1000), anti-HA (901514, Biolegend, 1:1000), anti-Myc (AB32, Abcam, 1:1000), anti-Actin (3700, Cell Signaling Technology, 1:1000), anti-RNF187 (HPA030098, Sigma, 1:1000), anti-P53 (SC126, Santa Cruz, 1:1000), and anti-cleaved Caspase-3 (9661, Cell Signaling Technology, 1:1000). Protein signals were detected with an ECL kit (Millipore Co., Billerica, MA, USA).

### Plasmids and siRNA

The Myc-RNF187 plasmid was acquired from Origene (RC229128). The P53 deletion constructs were used in a previous study and were provided by Dr. Yu as a gift [22]. The full-length and deletion constructs of RNF187 were subcloned from the full-length construct. The HA-K48 and HA-K63 Ubi plasmids were used in a previous study [41]. Plasmids were transfected with Lipofectamine 2000 (1662298, Invitrogen). The RNF187 and P53 siRNA sequences used for siRNA transfection are shown in Supplementary Table 1. The siControl sequences were 5′-UUCUCCGAACGUGUCACGUTT-3′ and 5′-ACGUGACACGUUCGGAGAATT-3′.

### Quantification of cell viability

MCF-7, MDA-MB-175, and ZR751 cells were transfected with siRNF187 or siControl in 24-well plates. Twenty-four hours after transfection, the cells were counted, and 4000 cells were seeded into 96-well plates. The relative cell viability was measured at the indicated time points. Cell numbers were determined using the CCK-8 cell proliferation reagent by measuring the absorbance at 450 nm. For the cisplatin-induced cell death assay, MDA-MB-175 cells were transfected with siRNF187 or siControl in 24-well plates. Twenty-four hours after transfection, the cells were counted, and 20,000 cells were seeded into 96-well plates. Cells were incubated with different concentrations of cisplatin for 24 h. Cell viability was measured using the CCK-8 cell proliferation reagent by measuring the absorbance at 450 nm.

### Flow cytometric analyses

For cell cycle analysis, MCF-7 cells were transfected with 50 nM siRNF187 or siControl. After 24 h, the cells were fixed with 70% ethanol and stained with propidium iodide. For apoptosis analysis, MDA-MB-175 cells were transfected with 50 nM siRNF187 or siControl. Twenty-four hours post transfection, the cells were stained with propidium iodide and annexin V. A BD LSR flow cytometer was used to measure the fluorescence intensity.

### Coimmunoprecipitation assay

Immunoprecipitation was performed as described in a previous study [42]. Total MCF-7 cell lysates were precleared with rabbit IgG for 2 h and subsequently immunoprecipitated with an anti-RNF187 antibody (HPA030098, Sigma) overnight, while rabbit IgG (Santa Cruz) was used as the negative control. Bound proteins were analyzed by western blotting with an anti-P53 antibody (SC126, Santa Cruz). For the overexpression experiment, HEK293 cells were cotransfected with 5 μg of GFP-RNF187 plasmid (full-length RNF187 or domain deletion mutants) and 5 μg of P53 plasmid. Cell lysates were precleared with IgG and subsequently incubated with an anti-P53 (SC126, Santa Cruz) antibody, while mouse IgG was used as the negative control. Bound proteins were analyzed by western blotting with an anti-GFP antibody (AB290, Abcam). Accordingly, the GFP-P53 plasmid (full-length P53 or domain deletion mutants) was cotransfected with 5 μg of Myc-RNF187 plasmid in 10 cm dishes. Cell lysates were precleared with IgG and subsequently incubated with an anti-Myc (AB32, Abcam) antibody, while rabbit IgG was used as the negative control. Bound proteins were analyzed by western blotting.

### SA-beta-gal activity

For the SA-beta-gal staining, MDA-MB-175 cells are utilized for cell senescence. The SA-beta-gal staining was measured according to the protocol of Senescence beta-Galactosidase Staining Kit (9860, Cell Signaling Technology). In general, MDA-MB-175 cells were transfected with 50 μM RNF187 siRNA or siControl. After that, the senescence cells were stained and counted at the indicated time points.

### Protein stability assays

Approximately 10^5^ MCF-7 cells were seeded into 24-well plates and transfected with 50 μM RNF187 siRNA or siControl. After 48 h, the cells were treated with 100 μM cycloheximide (C7698, Sigma) for the indicated times. Samples were subjected to western blotting to evaluate P53 degradation.

### Polyubiquitination assay

To directly detect enriched K48-ubiquitinated and total ubiquitinated P53 in cell extracts, HEK293 cells were transfected with the K48 Ubi, K63 Ubi, or Ub plasmid together with the GFP-P53 plasmid and Myc-RNF187 or Myc-vector. After 24 h, the cells were treated with 20 μM MG132 for 7 h; total protein was then extracted, and lysates were precleared with 30 μl of protein A (Santa Cruz, SC-2001) for 4 h. The supernatant was collected and immunoprecipitated with an anti-P53 antibody. Western blotting with an anti-HA antibody was performed to detect total polyubiquitinated P53 or K48/K63-polyubiquitinated P53.

### Ubiquitination assay of purified proteins

Ubiquitination was analyzed with a ubiquitination kit (Boston Biochem) following protocols recommended by the manufacturer. The recombinant proteins MDM2 and P53 were acquired from Sangon Biotech (China). The RNF187 protein was expressed in and purified from HEK293 cells with anti-Myc beads. Recombinant proteins were mixed with 20X E1 enzyme, 10X Mg^2+^-ATP solution, 10X ubiquitin solution, 1 μg E2 enzyme (UbcH7, Boston Biochem; UBE2D1, Sino Biological Inc.) in a final volume of 20 µl reaction buffer. The reaction was carried out at 37 °C for 1 h, and the products were analyzed by western blotting with an anti-P53 antibody (Santa Cruz, SC126).

### Immunofluorescence assay

MCF-7 cells were fixed with 4% paraformaldehyde in PBS for 10 min, permeabilized with 0.2% Triton X-100 for 5 min, and blocked with 5% BSA in PBS for 1 h. A rabbit anti-RNF187 (HPA030098, Sigma, 1:100) antibody and mouse anti-P53 monoclonal antibody (SC126, Santa Cruz, 1:100) were used as primary antibodies, followed by Alexa Fluor 647-conjugated (Invitrogen) anti-rabbit and FITC-conjugated anti-mouse antibodies (Jackson ImmunoResearch, West Grove, PA) as secondary antibodies. As negative controls, samples were incubated with secondary antibodies without the primary antibody incubation step. Images were acquired under conditions satisfying the Nyquist criterion using a Nikon A + laser scanning confocal microscope system with a 60X oil NA1.4 objective and pinhole size of 1.0 Airy unit. The acquired images were further processed and assembled using ImageJ.

### Xenograft tumor model

Stable knockdown cell lines were generated with MCF-7 cells, which were transduced with shControl or shRNF187 lentiviral vectors. The shRNF187 sequences were generated according to siRNF187#1 sequences. After 48 h of transduction, cells were selected with 1 μg/ml puromycin for 3 days. Female nonobese diabetic SCID mice were implanted with slow-release 17 beta-estradiol pellets (0.72 mg/90 days, Innovative Research of America). After 24 h, approximately one million MCF-7 cells together with Matrigel solution were injected into a mammary fat pad of each mouse. The tumor size was measured every 3 days. After 6 weeks, the mice were sacrificed, and the tumors were weighed and photographed. The experiments were performed under the protocols approved by the ethics committee of Xinxiang Medical University.

### Statistical analysis

No specific statistical tests were used to predetermine the sample size. Statistical analysis was performed using GraphPad Prism 7 software or SPSS version 23.0. Data were expressed as the mean ± s.e.m. values. Differences between two independent groups were evaluated with Student’s *t*-test. The Kaplan−Meier method with the log-rank test was applied for survival analysis. Differences were considered to be statistically significant when *P* < 0.05 (*P < 0.01; **P < 0.001).

## Supplementary information

Supplementary figure 1

Supplementary figure 2

Supplementary figure 3

Sequences for siRNA and QPCR

Supplementary figure legends

Reproducibility Checkist

Original Data File

Cell line authentication

Authorship change form

## Data Availability

The publicly available data are provided in the supplementary materials. The datasets used and analyzed during the current study are available from the corresponding author on reasonable request.
