# Supplementary figures and images for: Regulation of P53 signaling in breast cancer by the E3 ubiquitin ligase RNF187
Source: Cell Death Dis. 2022 Feb 14;13(2):149. doi: 10.1038/s41419-022-04604-3 (PMC8844070; doi:10.1038/s41419-022-04604-3)

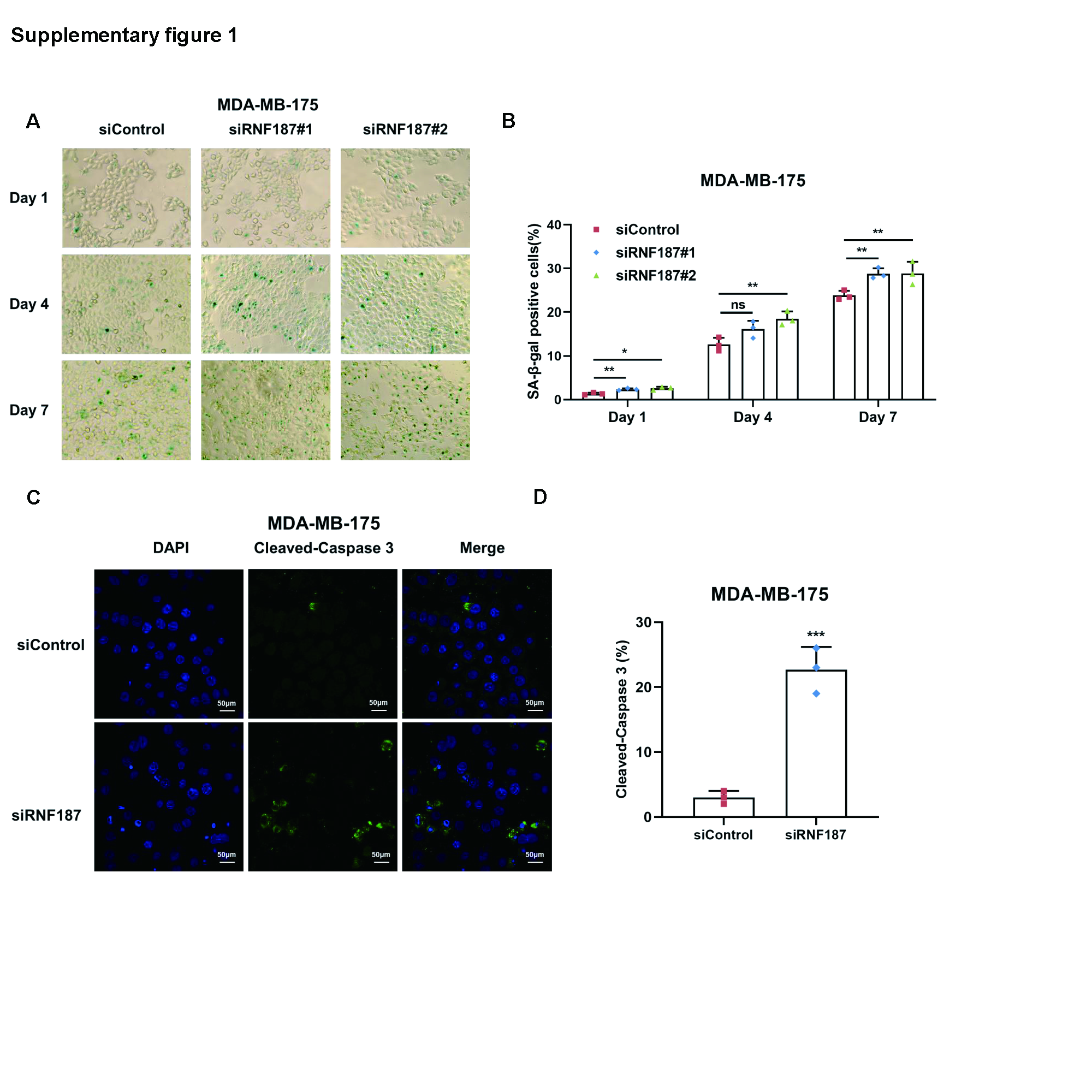

Supplement: Supplementary file 1 — Supplementary figure 1 [file 41419_2022_4604_MOESM1_ESM.tif]

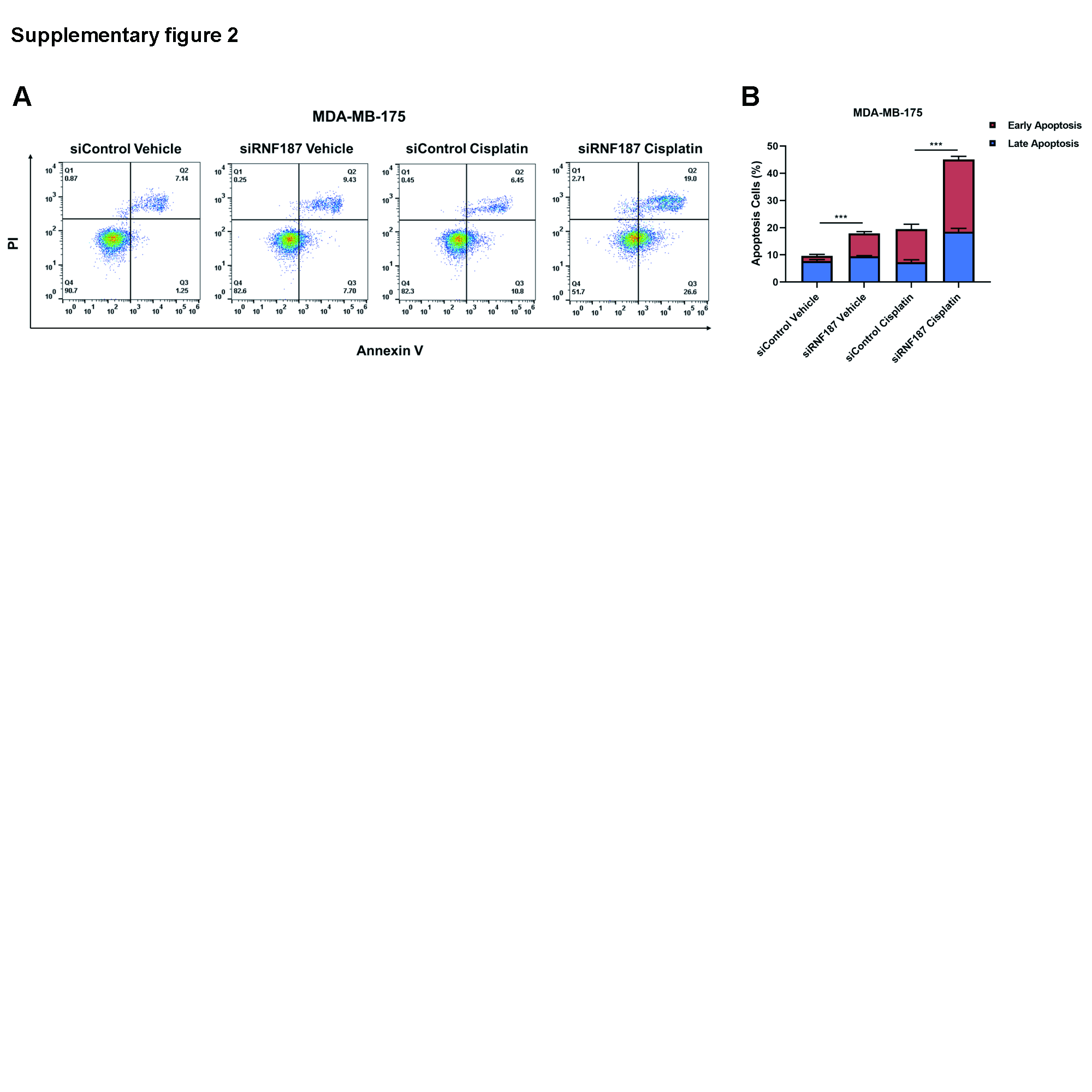

Supplement: Supplementary file 2 — Supplementary figure 2 [file 41419_2022_4604_MOESM2_ESM.tif]

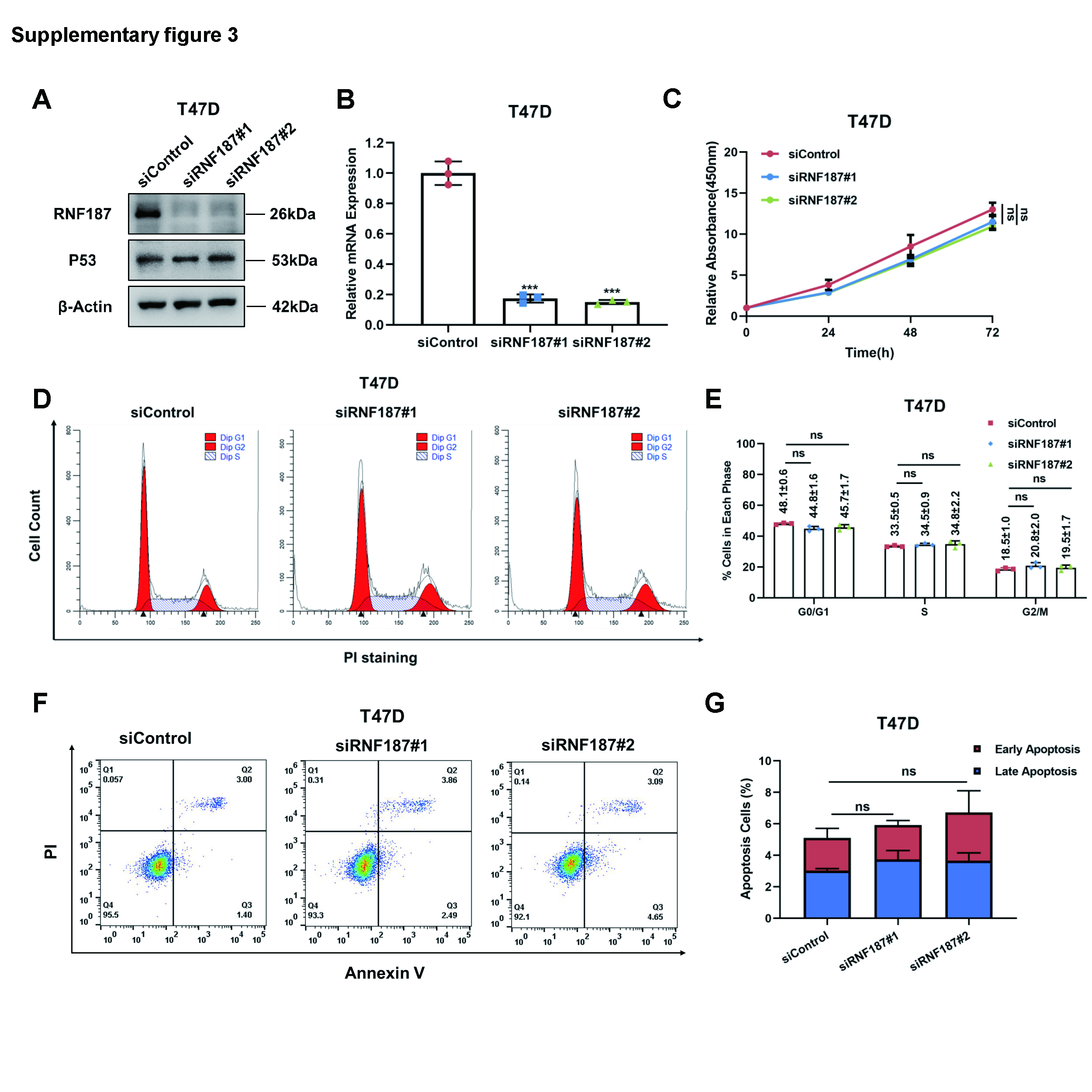

Supplement: Supplementary file 3 — Supplementary figure 3 [file 41419_2022_4604_MOESM3_ESM.tif]
