# Supplementary material for: Regulation of P53 signaling in breast cancer by the E3 ubiquitin ligase RNF187
Source: Cell Death Dis. 2022 Feb 14;13(2):149. doi: 10.1038/s41419-022-04604-3 (PMC8844070; doi:10.1038/s41419-022-04604-3)
Supplement: Supplementary file 5 — Supplementary figure legends [file 41419_2022_4604_MOESM5_ESM.docx]

**Supplementary Figure 1: RNF187 depletion enhances cellular senescence and increases apoptosis**

**A-B:** RNF187 depletion could significantly enhance breast cancer cell senescence. MDA-MB-175 cells were transfected with siControl or siRNF187. The beta-Gal staining was performed at 1, 4 and 7 days. Each group was analyzed in triplicate. *P<0.05; ** P<0.01; ***P<0.001 for comparisons.

**C-D:** RNF187 depletion significantly increased the number of caspase-3 positive cells. MDA-MB-175 cells were transfected with siControl or siRNF187. After 48 hours, immunofluorescence staining of the cells was performed. Cleaved-caspase 3 (Green) is shown. Nuclei (blue) were stained with 4’,6-diamidino-2-phenylindole (DAPI). Each group was analyzed in triplicate. *P<0.05; ** P<0.01; ***P<0.001 for comparisons.

**Supplementary Figure 2: RNF187 depletion increased the proportion of apoptotic cells, which effect could be further enhanced by cisplatin treatment**

**A-B:** MDA-MB-175 cells were transfected with siControl or siRNF187. After 48 hours, cells were treated with vehicle or 10 uM cisplatin. After 6 hours, cells were stained with PI and Annexin V. Then cells were subject to FACS analysis for the proportion of apoptotic cells. Each group was done in triplicates. *P<0.05; ** P<0.01; ***P<0.001 for comparisons.

**Supplementary Figure 3: RNF187 is non-essential for cancer cell growth and anti-apoptosis in P53 mutant/luminal breast cancer**

**A-B:** T47D cells were transfected with siControl or siRNF187. After 48 hours, the cells were harvested for western blot analysis. RNF187 and P53 protein levels were determined by western blot analysis. Actin was used as the internal control. Each group was analyzed in triplicate. *P<0.05; ** P<0.01; ***P<0.001 for gene expression comparisons.

**C:** T47D cells were transfected with siControl or siRNF187. Two independent siRNAs were used. After 24 hours, a CCK-8 assay was used to determine the cellular metabolic activity at the indicated time points after transfection. Experiments were performed in triplicate. *P<0.05; ** P<0.01; ***P<0.001 for cell growth comparisons.

**D-E:** Cell cycle analysis to assess the effect of RNF187 knockdown in T47D cells. T47D were transfected with siControl or siRNF187. Two independent siRNAs were used. After 24 hours, the cells were harvested, fixed with 70% ethanol and stained with propidium iodide. The cells were subjected to FACS analysis. Experiments were performed in triplicate. *P<0.05; ** P<0.01; ***P<0.001 for cell proportion comparisons.

**F-G:** T47D cells were transfected with siControl or siRNF187. After 48 hours, cells were stained with PI and Annexin V. Then cells were subject to FACS analysis for the proportion of apoptotic cells. Each group was done in triplicates. *P<0.05; ** P<0.01; ***P<0.001 for comparisons.
