# Supplementary material for: Regulation of P53 signaling in breast cancer by the E3 ubiquitin ligase RNF187
Source: Cell Death Dis. 2022 Feb 14;13(2):149. doi: 10.1038/s41419-022-04604-3 (PMC8844070; doi:10.1038/s41419-022-04604-3)
Supplement: Supplementary file 7 — Original Data File [file 41419_2022_4604_MOESM7_ESM.pdf]

Figure 1E

Time(h)	siControl			siRNF187#1			siRNF187#2		
0	0.997802	0.993407	1.008791	1.079727	0.958998	0.961276	1.088847	0.952741	0.960302
24	5.512088	5.784615	5.096703	3.57631	3.553531	3.46697	3.018904	2.89225	2.924386
48	14.84835	13.61538	15.94505	7.826879	9.330296	9.277904	5.975425	6.517958	7.047259
72	18.01978	19.05714	19.53846	12.79954	13.84282	14.01139	10.99055	10.42911	11.55577

Figure 1F

Time(h)	siControl			siRNF187#1			siRNF187#2		
0	0.876834	1.040389	1.082792	0.979312	0.932752	1.087952	1.065914	0.862797	1.071307
24	4.874835	4.023346	4.803659	2.70824	3.758527	3.287135	2.794625	2.820269	3.420632
48	13.46741	13.94909	13.08412	8.542671	7.943135	8.037807	6.679489	7.622332	7.233117
72	17.99079	17.06065	17.00295	9.729486	10.07248	9.065439	7.97728	7.073981	8.106699

Figure 1G

Time(h)	siControl			siRNF187#1			siRNF187#2		
0	0.906588	1.087906	1.005489	0.914443	1.010701	1.074872	0.914443	1.010701	1.074872
24	5.431289	5.497223	5.068653	2.600552	3.096809	3.296809	2.719266	3.096809	2.655094
48	8.2472	9.999951	9.27468	3.518202	3.834245	4.555102	3.799488	3.27596	3.255102
72	14.96146	13.79114	13.4285	7.272217	7.156174	7.936388	5.65779	6.571161	6.042818

Figure 1L

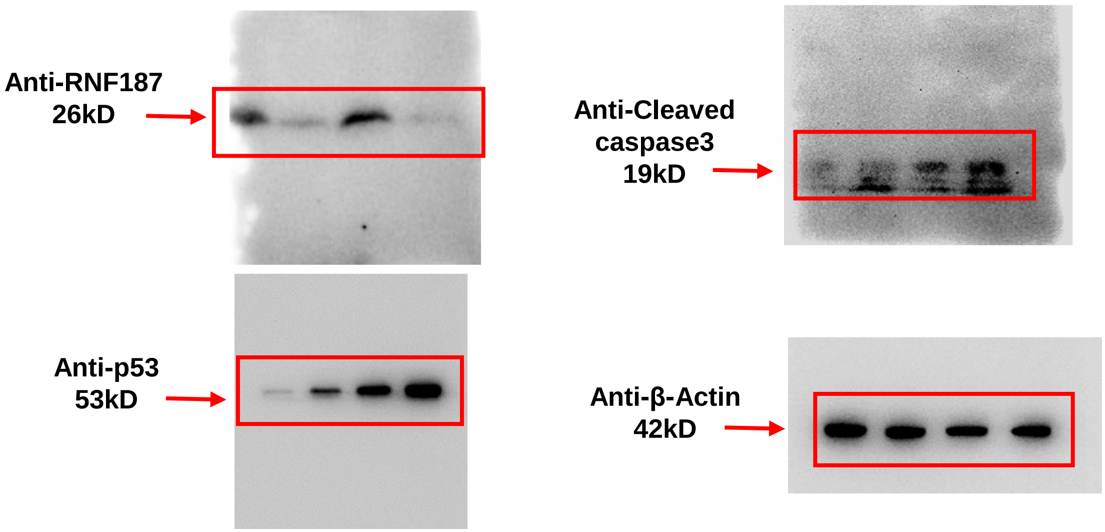

Anti-RNF187
26kD
Anti-Cleaved
caspase3
19kD
Anti-p53
53kD
Anti-β-Actin
42kD

Figure 1M

Cisplatin(μM)	siControl			siRNF187		
0	0.997738	1.013575	0.988688	0.959415	1.005681	1.034902
8	0.911765	0.9819	0.900452	0.762175	0.854707	0.896103
16	0.760181	0.841629	0.825792	0.513473	0.498863	0.436688
24	0.640271	0.696833	0.723982	0.38198	0.335551	0.320941
32	0.588235	0.717195	0.656109	0.287889	0.225454	0.215714
40	0.579186	0.508597	0.447059	0.128052	0.187013	0.127532

Figure 3A

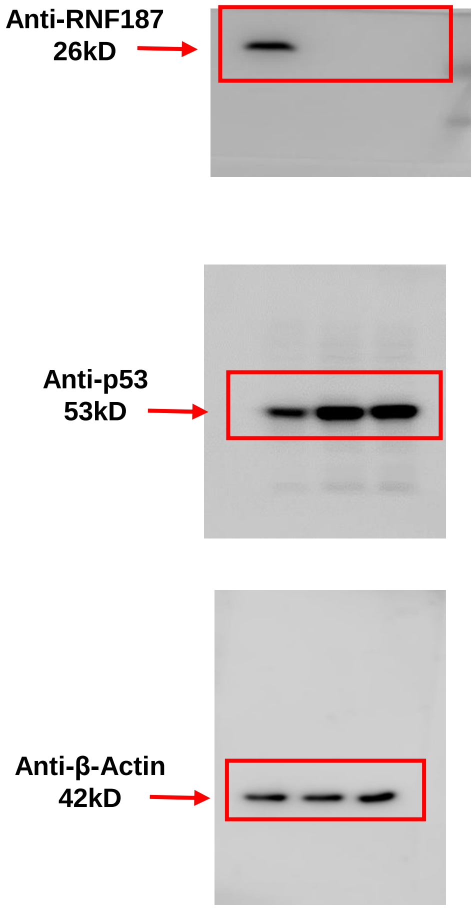

Figure 3C

	siControl			siRNF187#1			siRNF187#2		
P53INP1	1.059496	0.915138	1.025197	4.239294	4.210344	3.748905	3.787398	2.916964	3.29404
P21	1.065213	0.996435	0.938465	2.581142	3.170254	3.424896	4.03974	5.30264	5.2457
BTG2	0.965079	1.001775	1.032704	3.347953	2.448265	2.560149	4.945892	5.02047	5.460678
BAX	0.984316	0.994979	1.020656	3.065587	2.978539	2.820568	3.827283	3.441747	3.665992

Figure 3D

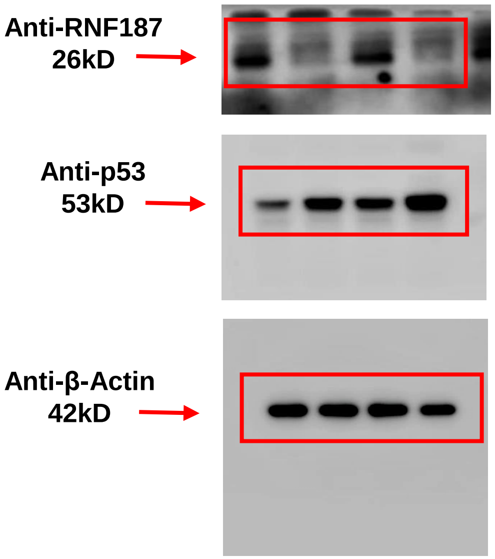

Figure 3E

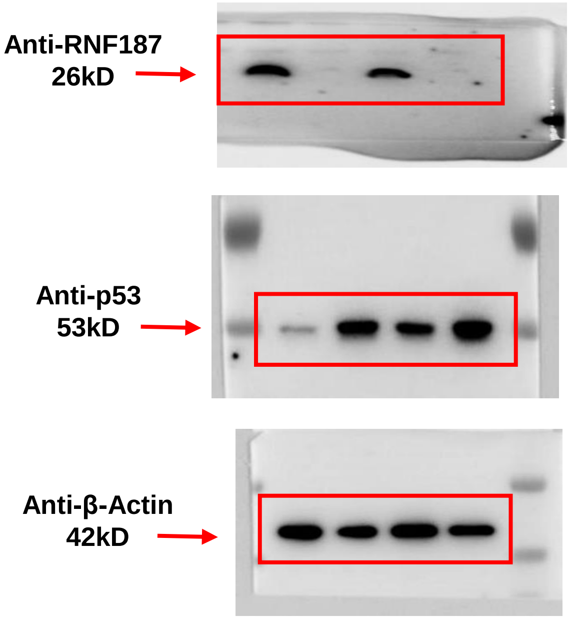

Figure 3F

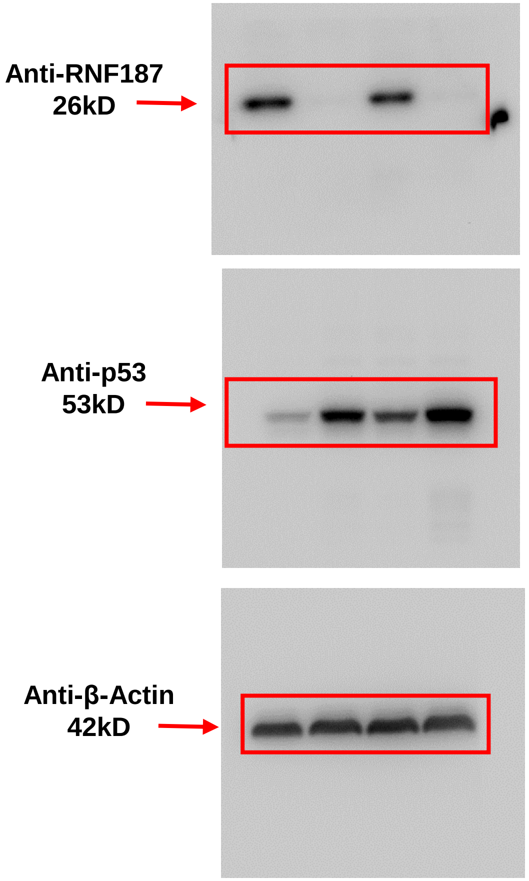

Figure 3G

	siControl+Vehicle			siRNF187+Vehicle			siControl+Cisplatin			siRNF187+Cisplatin		
P53INP1	1.009345	0.945824	1.044323	2.207663	2.12139	2.145905	2.661142	2.448007	2.6681	3.85178	3.813115	3.887684
P21	1.089362	1.005184	0.905442	2.259027	2.375903	2.580061	5.483117	5.611781	4.836528	9.86715	8.364579	8.021797
BTG2	1.003413	0.977975	1.018375	3.2013	3.130099	3.180334	3.393154	3.486725	3.578999	5.832558	5.990349	6.02134
BAX	1.08677	1.026162	0.887156	2.435925	2.314966	2.650207	2.204112	2.205415	2.125169	4.04383	4.088491	4.07262

Figure 3H

	siControl+Vehicle			siRNF187+Vehicle			siControl+Cisplatin			siRNF187+Cisplatin		
P53INP1	1.022256	1.022322	0.955653	8.376135	8.496781	7.774995	2.288713	2.237563	2.119314	10.50461	10.04416	10.63484
P21	0.911369	1.073152	1.015503	4.102641	3.690866	3.259424	2.334243	2.230405	2.302885	11.54561	9.652185	9.725313
BTG2	1.05639	0.966567	0.97713	5.11737	4.165703	4.240725	2.001815	1.957957	1.867986	5.903262	5.867633	5.870046
BAX	0.990161	1.03614	0.973687	2.269149	2.139396	2.089649	1.780737	1.748049	1.813424	8.38568	7.789322	7.764153

Figure 3I

	siControl+Vehicle			siRNF187+Vehicle			siControl+Cisplatin			siRNF187+Cisplatin		
P53INP1	0.970203	1.085027	0.94477	1.93498	1.76653	1.917415	2.425986	2.958953	2.912351	4.455044	4.611294	4.510123
P21	1.103026	0.903503	0.993471	3.290732	4.741678	3.651392	4.579904	4.565549	5.686492	7.901556	8.116201	8.212242
BTG2	1.048922	1.046612	0.904466	4.088145	5.401432	4.172018	3.124812	2.699217	3.586869	7.294384	7.026983	6.69265
BAX	1.011771	1.01907	0.969161	1.629523	2.20748	1.700421	1.528955	1.755337	1.788532	3.194541	3.463812	3.191474

Figure 4A

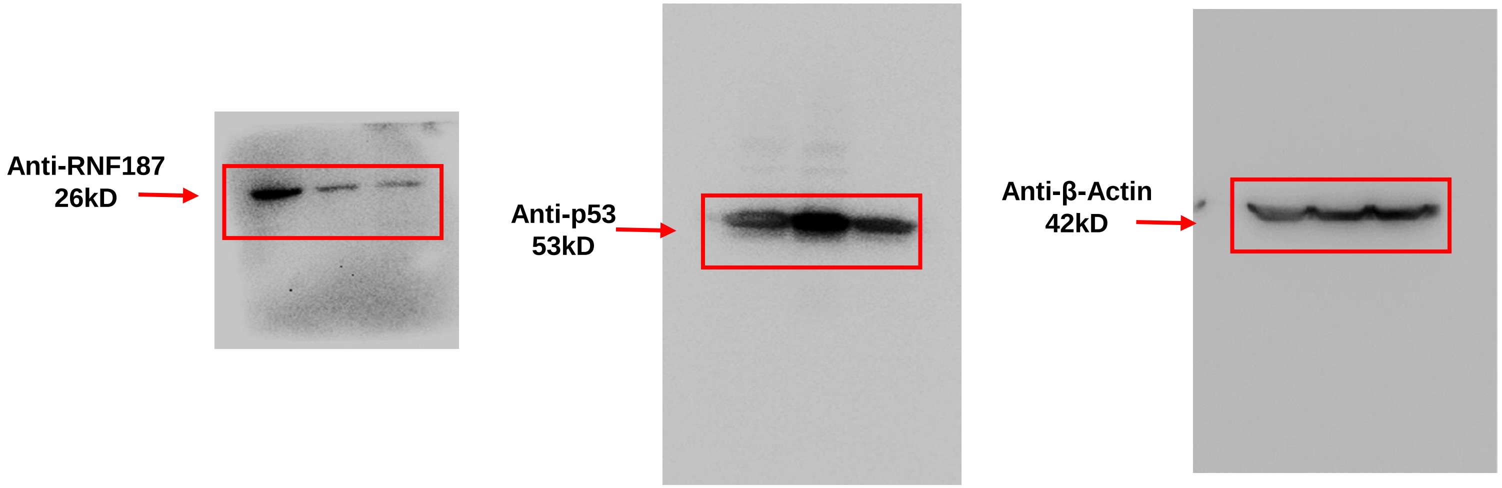

Figure 4B

	siControl+siControl			siRNF187+siControl			siRNF187+siP53		
BTG2	0.966021	1.011139	0.985139	1.84288	2.02803	2.213181	1.055961	1.019476	1.32231
BAX	0.791689	1.124834	1.012874	2.138436	2.088758	2.57721	1.455227	1.339088	1.299747
P21	0.984431	0.92889	1.054992	2.911658	2.407338	2.659498	1.312304	1.19282	1.089888

Figure 4C

Time(h)	siControl+siControl			siRNF187+siControl			siRNF187+siP53		
0	0.979664	0.905377	1.114973	0.933897	1.106072	0.960031	0.948364	1.038484	1.013138
24	4.952045	4.814083	5.202765	1.775557	1.429669	1.114374	2.973936	3.275272	3.986369
48	9.023255	8.39492	9.252749	2.634896	2.934743	2.198155	6.965938	6.87441	5.945055
72	14.32949	13.04803	13.23243	4.672175	3.752498	3.935434	9.782165	9.690637	8.761283

Figure 5B

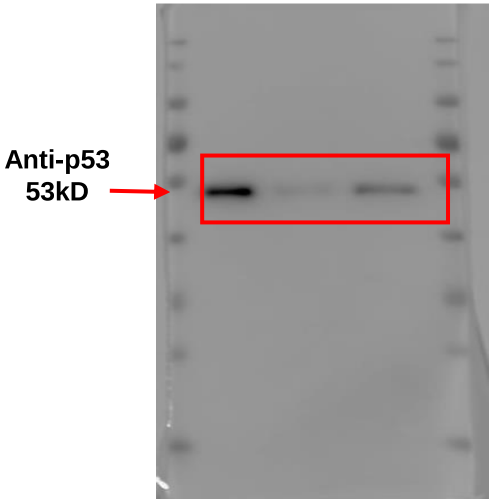

Figure 5C

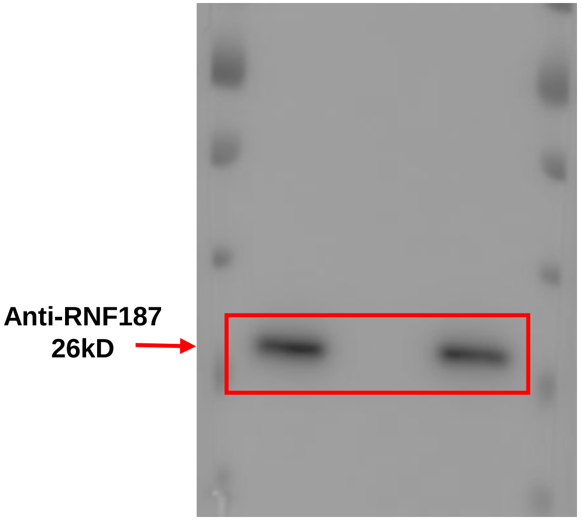

Figure 5D

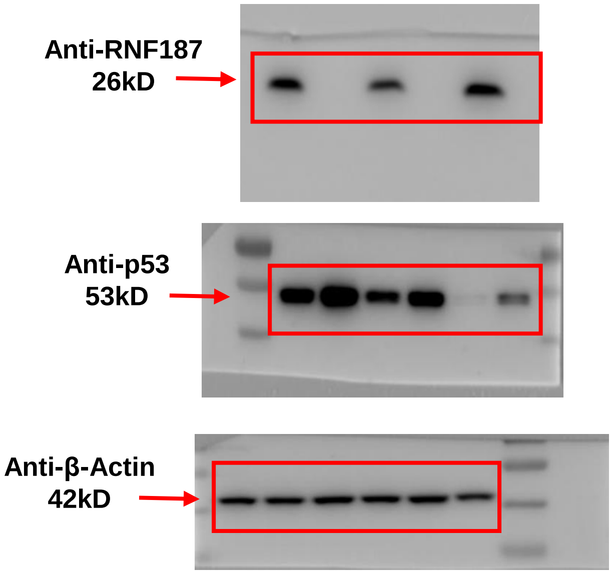

Figure 5E

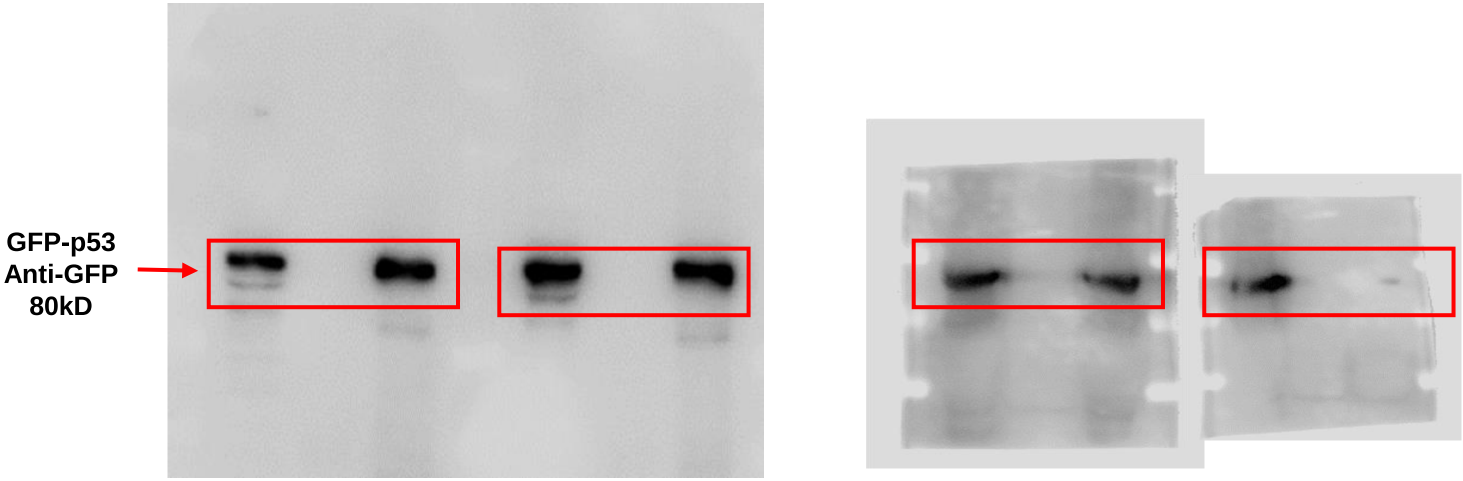

Figure 5G

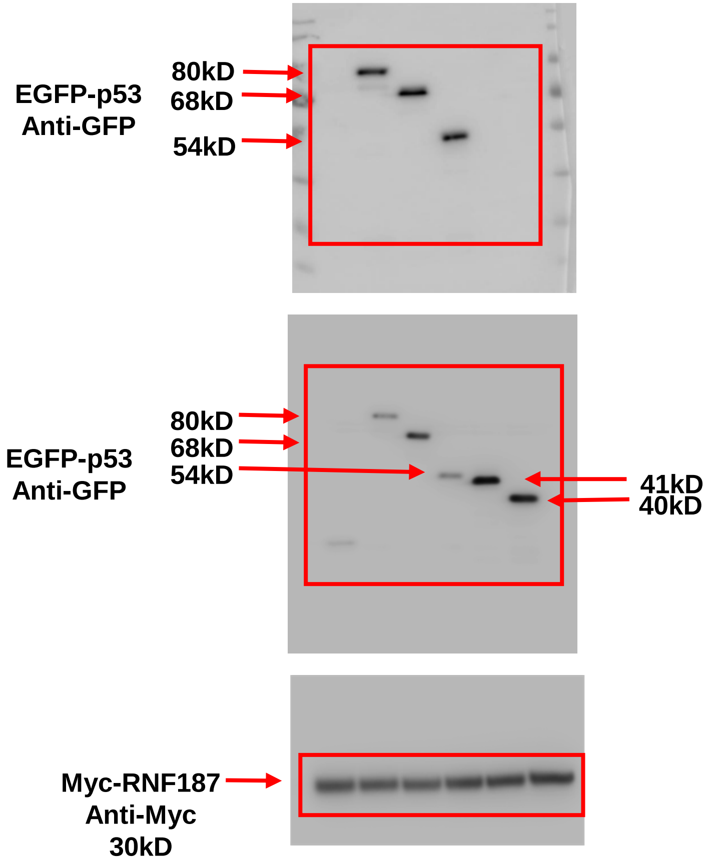

EGFP-p53
Anti-GFP
80kD
68kD
54kD
EGFP-p53
Anti-GFP
80kD
68kD
54kD
41kD
40kD
Myc-RNF187
Anti-Myc
30kD

Figure 5H

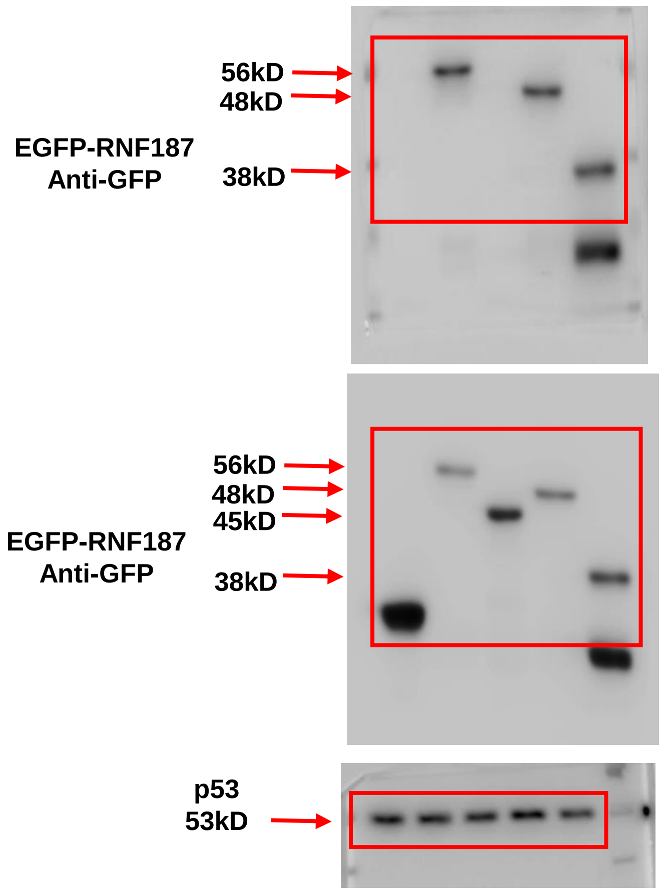

56kD
48kD
EGFP-RNF187
Anti-GFP
38kD
56kD
48kD
45kD
EGFP-RNF187
Anti-GFP
38kD
p53
53kD

Figure 6A

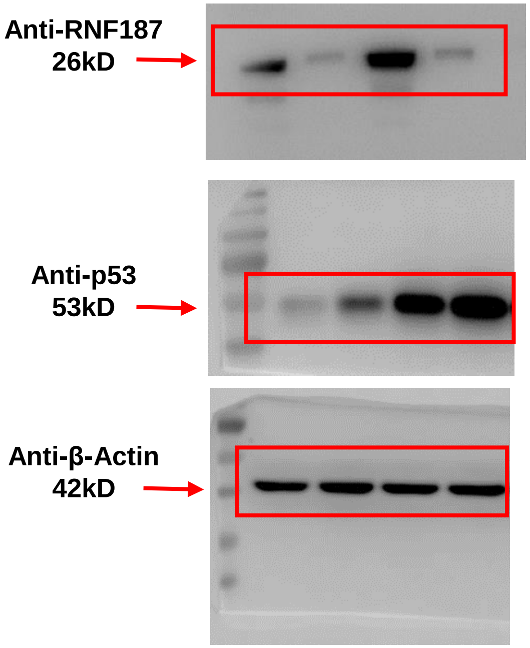

Figure 6B

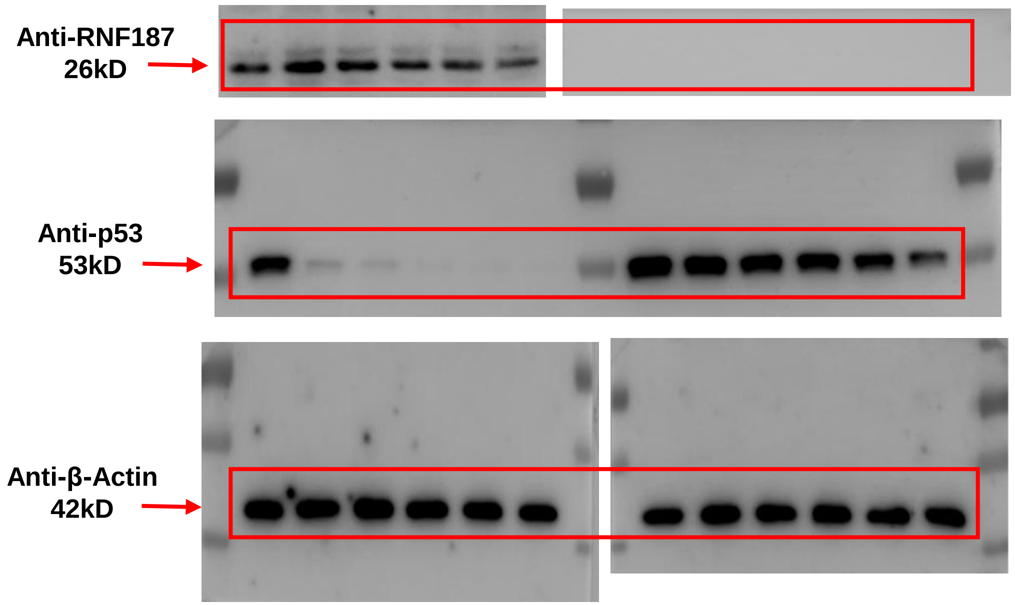

Figure 6D

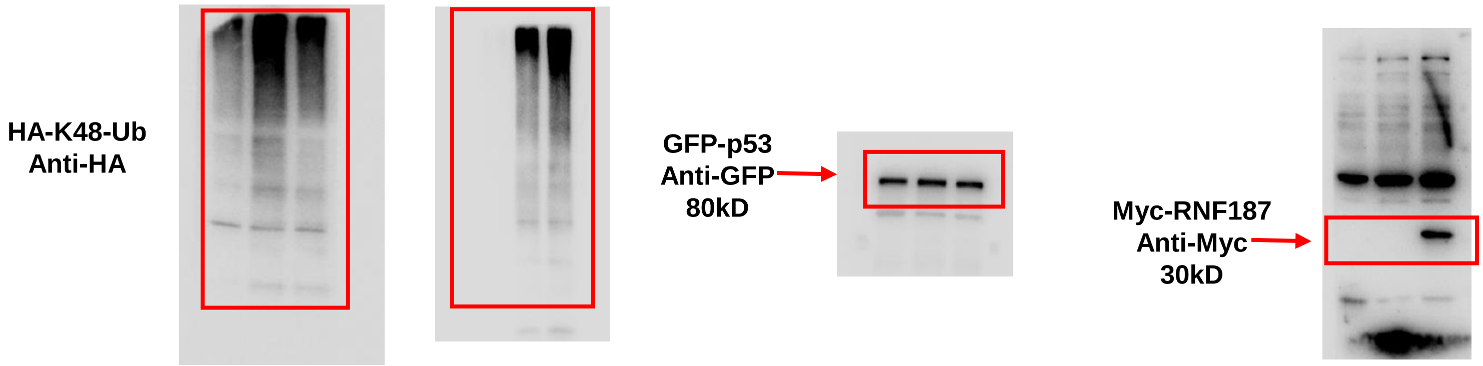
HA-K48-Ub
Anti-HA
GFP-p53
Anti-GFP
80kD
Myc-RNF187
Anti-Myc
30kD

Figure 6E

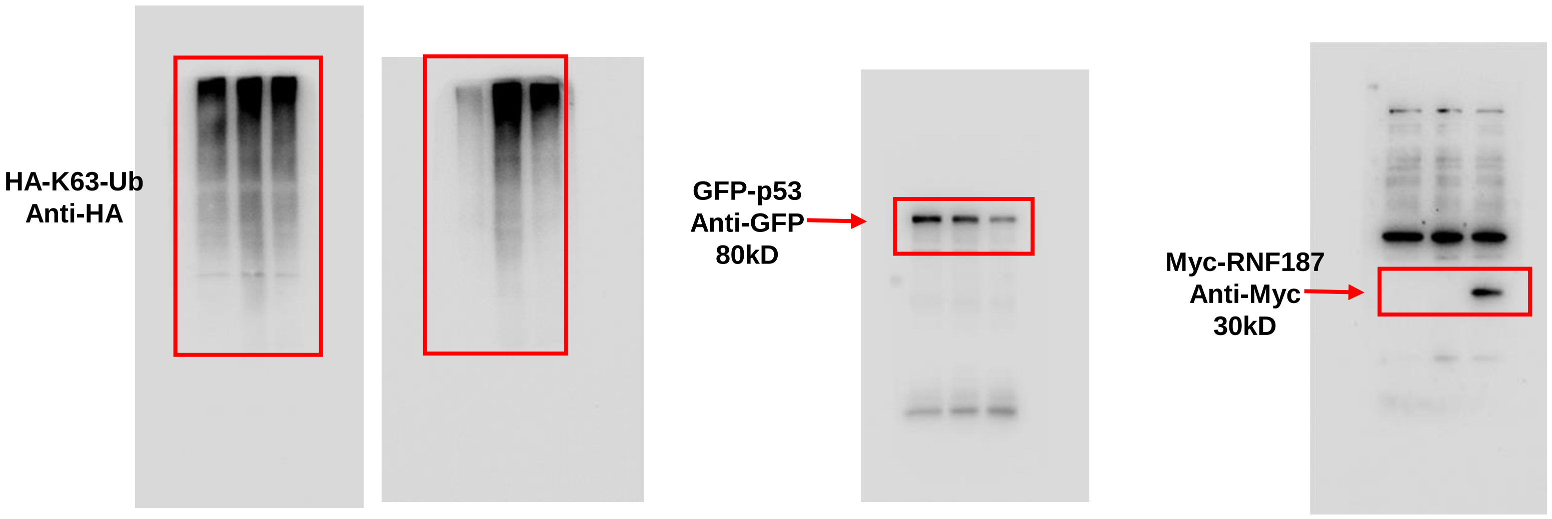
HA-K63-Ub
Anti-HA
GFP-p53
Anti-GFP
80kD
Myc-RNF187
Anti-Myc
30kD

Figure 6F

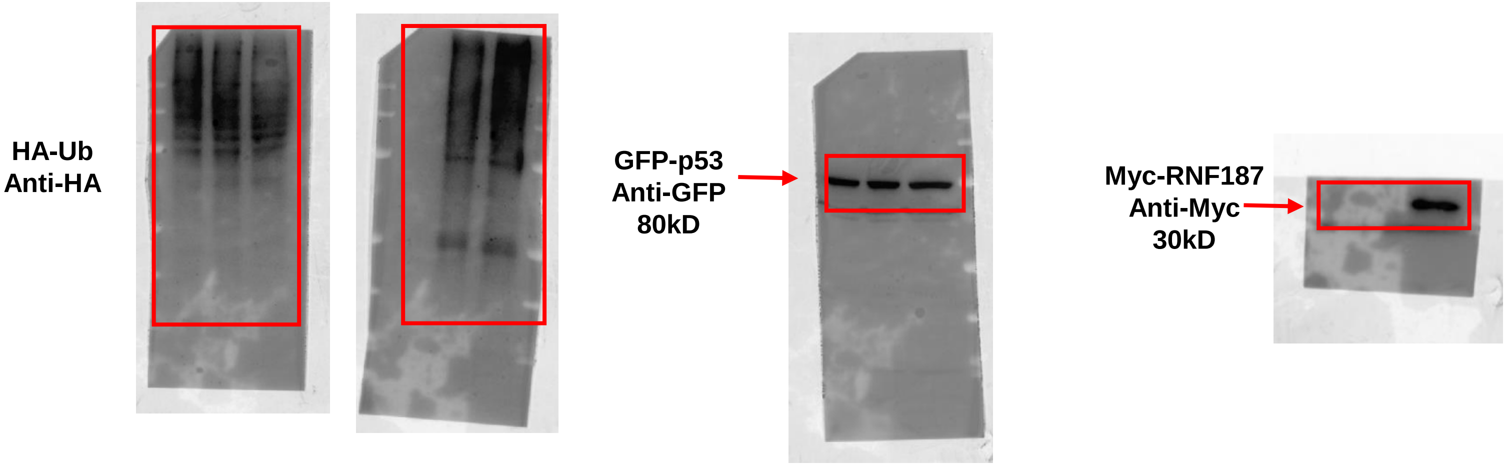

Figure 6H

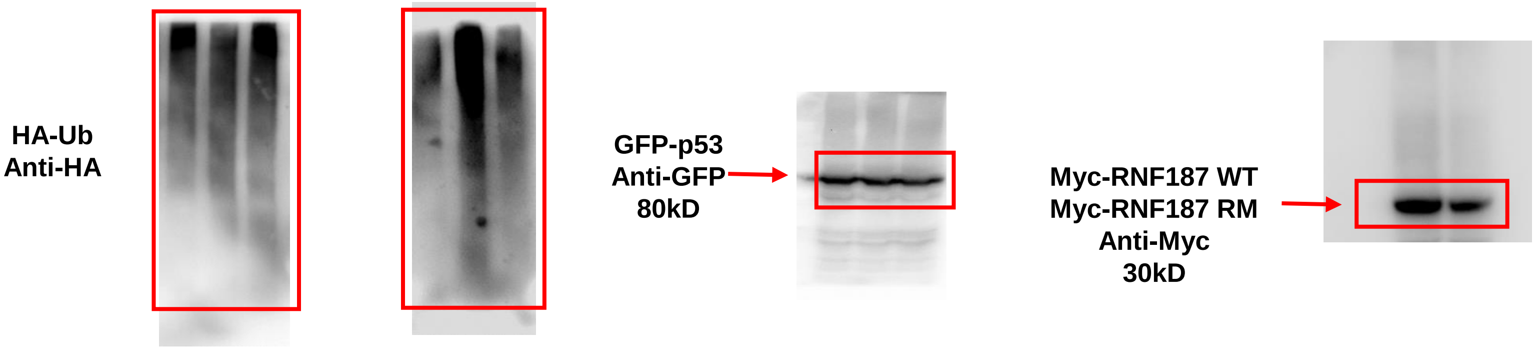

HA-Ub
Anti-HA
GFP-p53
Anti-GFP
80kD
Myc-RNF187 WT
Myc-RNF187 RM
Anti-Myc
30kD

Figure 6I

P53

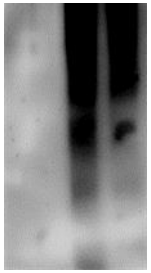

Flag-RNF187
Anti-Flag
30kD

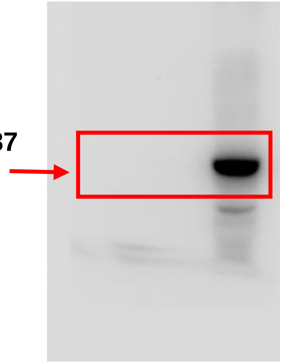

Supplementary Figure 3A

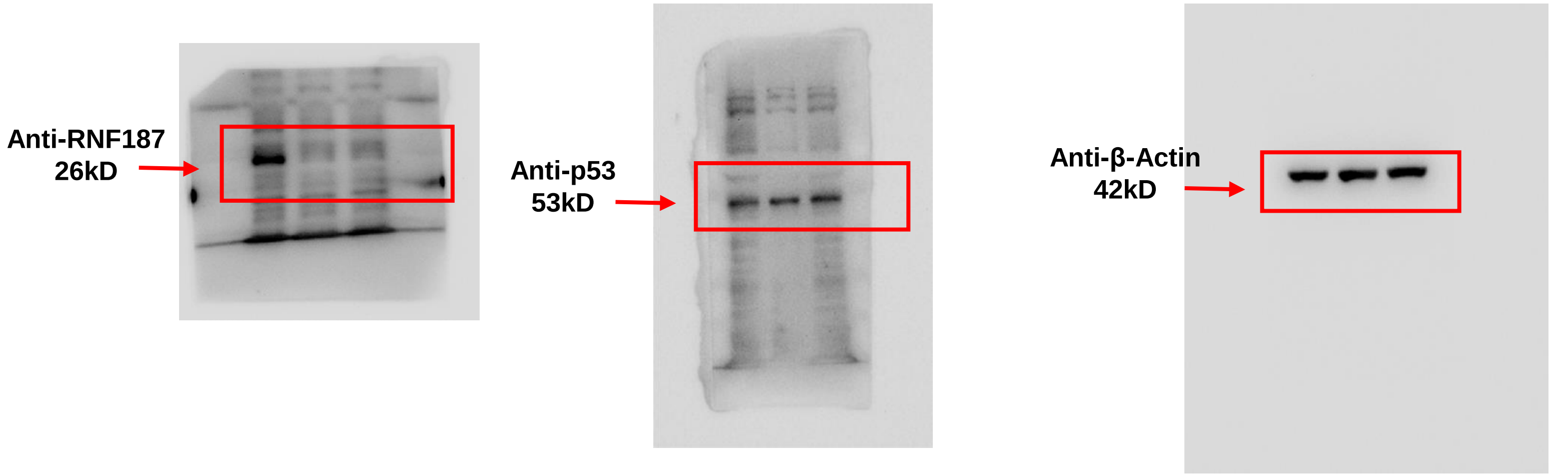

Supplementary Figure 3C

Time(h)	siControl			siRNF187#1			siRNF187#2		
0	1.198083	0.753994	1.047923	1.197026	0.929368	0.873606	0.716157	1.358079	0.925764
24	3.102236	4.166134	4.159744	3.092937	2.594796	2.992565	2.541485	2.982533	3.052402
48	7	9.776358	8.715655	6.32342	7.100372	7.327138	6.676856	6.131004	7.340611
72	12.63791	13.95101	12.41427	12.36307	11.42999	10.66791	10.60116	10.77584	11.43086
