# Supplementary material for: Regulation of P53 signaling in breast cancer by the E3 ubiquitin ligase RNF187
Source: Cell Death Dis. 2022 Feb 14;13(2):149. doi: 10.1038/s41419-022-04604-3 (PMC8844070; doi:10.1038/s41419-022-04604-3)
Supplement: Supplementary file 8 — Cell line authentication [file 41419_2022_4604_MOESM8_ESM.pdf]

Report of Human Cell Line Authentication

Delivery Date: November 06th, 2017

Analysis Date: November 13th, 2017

Ⅰ.Sample

Sample Name: ‘XB6568’, labeled as ‘MCF-7’, was received on November 06th, 2017.

Ⅱ.Methodand Procedure

1. PCR is amplified with STR Multi-amplification Kit(Microreader™21 ID System);
2. PCR products are assayed with ABI 3730xl DNA Analyzer(Applied Biosystems®).
3. Data were analyzed using GeneMapper3.2 software and then compared with the ATCC and DSMZ databases for reference matching.

III. Results

1. The results of the negative and positive control match expectations.
2. The STR profiles of the cell line sample are in the attached table and figure.

Genetic Site	ATCC				Customer sample				
(Locus)	Database profile：MCF7Breast AdenocarcinomaHuman				Query profile：MCF-7				
Amelogenin	X				X				
D5S818	11	12			11	12			
D13S317	11				11				
D7S820	8	9			8	9			
D16S539	11	12			11	12			
vWA	14	15			14	15			
TH01	6				6				
TPOX	9	12			9	12			

CSF1PO	10				10				
Number of shared alleles between query sample and database profile									14
Total number of alleles in the database profile									14
Percent match between the query and the database profile：									100%

MCF-7: ①No cross-contamination of other human cell line is found.②The submitted profile is an 100% match for the following ATCC human cell line(s) in the ATCC STR database (8 core loci plus Amelogenin): MCF7Breast AdenocarcinomaHuman. 100% matched cell lines are found in DSMZ data bank. And the cell line named as MCF7 *etc*.

Operator: Rui Wang　　　　Auditor: Bingjun Wu

Beijing Microread Genetics Co., Ltd

Notes：

1. Based on the ANSI Standard, cell lines with ≥80% match are considered to be related; i.e.,derived from a common ancestry. Cell lines with between a 55% to 80% match require further profiling for authentication of relatedness.

2. The short tandem repeat (STR) profile generated by Beijing Microread Genetics Co., Ltd is indicative only of the sample sent to Beijing Microread Genetics Co., Ltd at the time it was sent. This data and analysis are for research use only.

Table: STR profiles of MCF-7 cell line

Cell line MCF-7（Fig.XB6568）		
Marker	Allele 1	Allele 2
D19S433	13	14
D5S818	11	12
D21S11	30	30
D18S51	14	14
D6S1043	12	18
AMEL	X	X
D3S1358	16	16
D13S317	11	11
D7S820	8	9
D16S539	11	12
CSF1PO	10	10
Penta D	12	12
D2S441	10	14
vWA	14	15
D8S1179	10	14
TPOX	9	12
Penta E	7	12
TH01	6	6
D12S391	18	20
D2S1338	21	23
FGA	23	25

Figure: STR profiles of MCF-7 cell line

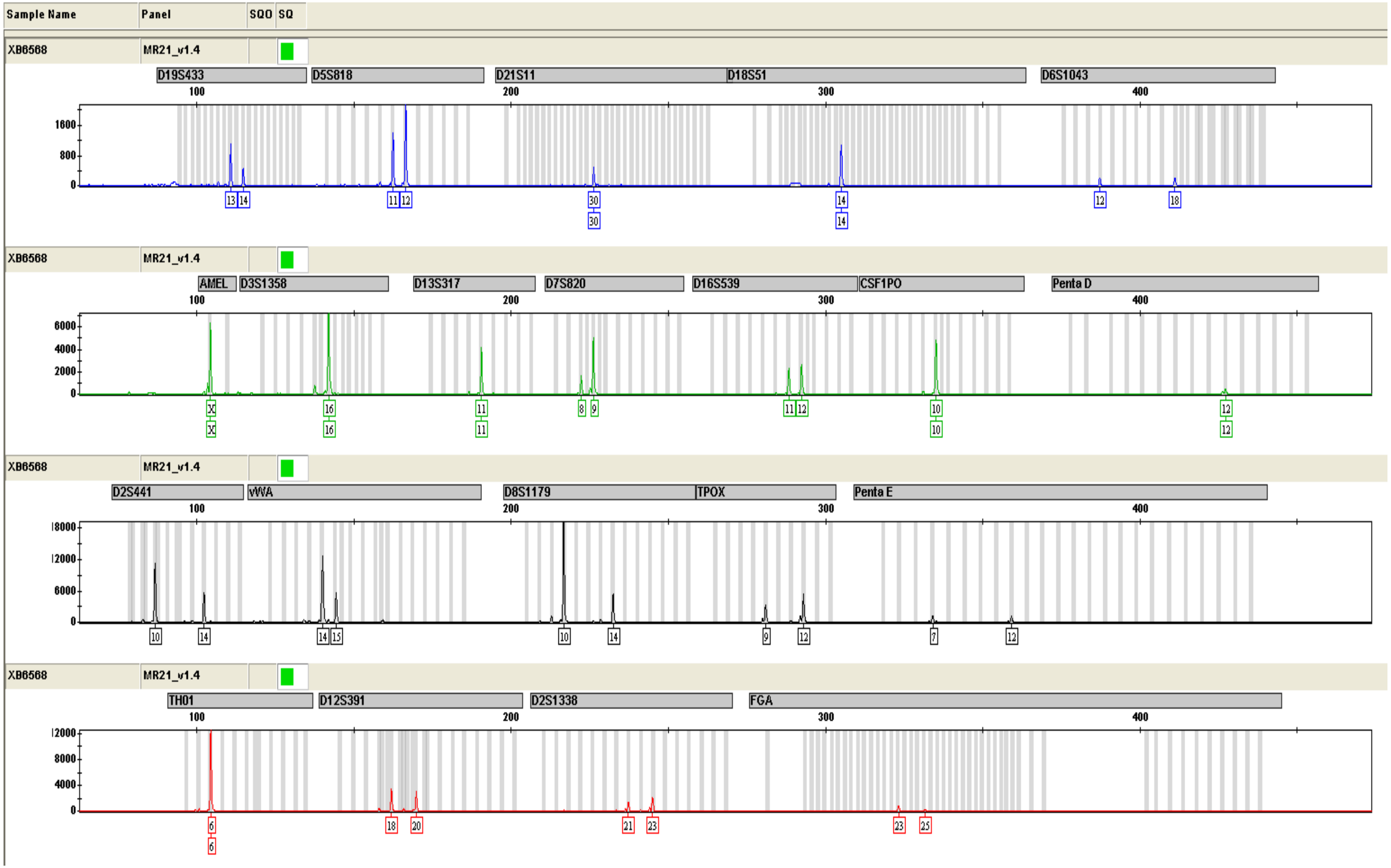

细胞 STR 检验报告

委托方：新乡医学院检验学院

委托日期：2017 年 11 月 06 日

鉴定日期：2017 年 11 月 13 日

一、 检材：

2017 年 11 月 06 日收到委托人 1 株细胞悬液：

编号为“XB6570”，包装上标记为“HEK293”。

要求鉴定该株细胞是否为单一来源细胞系, 是否存在交叉污染现象。

二、 检材处理和检验方法：

取适量检材用 Microread Genomic DNA Kit 提取 DNA，采用 Microreader^{TM}21 ID System 扩增 20 个 STR 位点和性别鉴定位点，使用 ABI 3730xl 型遗传分析仪进行 PCR 产物检测，使用 GeneMapper3.2 软件(Applied Biosystems)对检测结果进行分析，并与 ATCC 和 DSMZ 数据库进行比对。

三、 检验结果：

实验中阴性及阳性对照结果均正确。

该细胞株的 STR 位点和 Amelogenin 位点的基因分型结果见附表，分型图谱见附图。

Genetic Site	ATCC				检测样品				
(Locus)	数据库名称：HEK-293.2susEmbryonic KidneyHuman				样品名称: HEK293				
Amelogenin	X				X				
D5S818	8				8				
D13S317	12	14			12	14			
D7S820	11	12			11				
D16S539	9	13			9	13			
vWA	16	19			16	19			
TH01	7	9.3			7	9.3			
TPOX	11				11				

CSF1PO	12				12				
所有匹配峰的个数									13
ATCC 数据库所有峰的个数									14
匹配度									93%

四、 分析说明：

该细胞 DNA 扩增后图谱清晰，分型结果良好。

五、 检验结论：

HEK293:①该株细胞 DNA 进行细胞 STR 分型结果显示，在各基因座均未出现三等位基因现象。细胞中没有发现人类细胞交叉污染。② HEK293 细胞与 ATCC 中 HEK-293.2susEmbryonic KidneyHuman 细胞的 STR 数据匹配率为 93%，推测此样品为 HEK-293.2susEmbryonic KidneyHuman 的衍生细胞。③该株细胞 DNA 分型在 DSMZ 细胞库中未找到与其细胞分型 100%相匹配的细胞。

（此结果仅对本次检材负责）

操作人：王蕊　　　　　　　　审核人：吴炳君

北京阅微基因技术有限公司

备注：

1. 根据 ANSI 制定的国际标准，细胞系的匹配度≥80% 时，认为它们具有相关性，即衍生于共同的祖先细胞；匹配度在 55% 至 80% 之间，需要进一步验证相关性；小于 55%，表明两者不具有相关性。
2. 有效峰为真实的 PCR 条带;小峰和非特异性条带在计算中忽略不计。

本实验依照 Microreader™21 ID System STR 试剂盒提供的实验手段和分析方法进行检测，结果仅供参考。

Standards for Cell Line Authentications

To standardize STR analysis for human cell line authentication, the American Tissue Culture Collection (ATCC) Standards Development Organization Workgroup published ASN-0002-2011, which recommends the use of at least eight STR loci (TH01, TPOX, vWA, CSF1PO, D16S539, D7S820, D13S317 and D5S818) plus Amelogenin for gender identification for human cell line authentication.

附表：细胞 HEK293 的 STR 位点和 Amelogenin 位点的基因分型结果

细胞 HEK293（图片编号 XB6570）		
Marker	Allele 1	Allele 2
D19S433	15	18
D5S818	8	8
D21S11	30.2	30.2
D18S51	17	17
D6S1043	11	11
AMEL	X	X
D3S1358	15	17
D13S317	12	14
D7S820	11	11
D16S539	9	13
CSF1PO	12	12
Penta D	9	9
D2S441	11	15
vWA	16	19
D8S1179	12	14
TPOX	11	11
Penta E	7	15
TH01	7	9.3
D12S391	19	21
D2S1338	19	19
FGA	23	23

附图：细胞 HEK293 的 STR 分型图谱

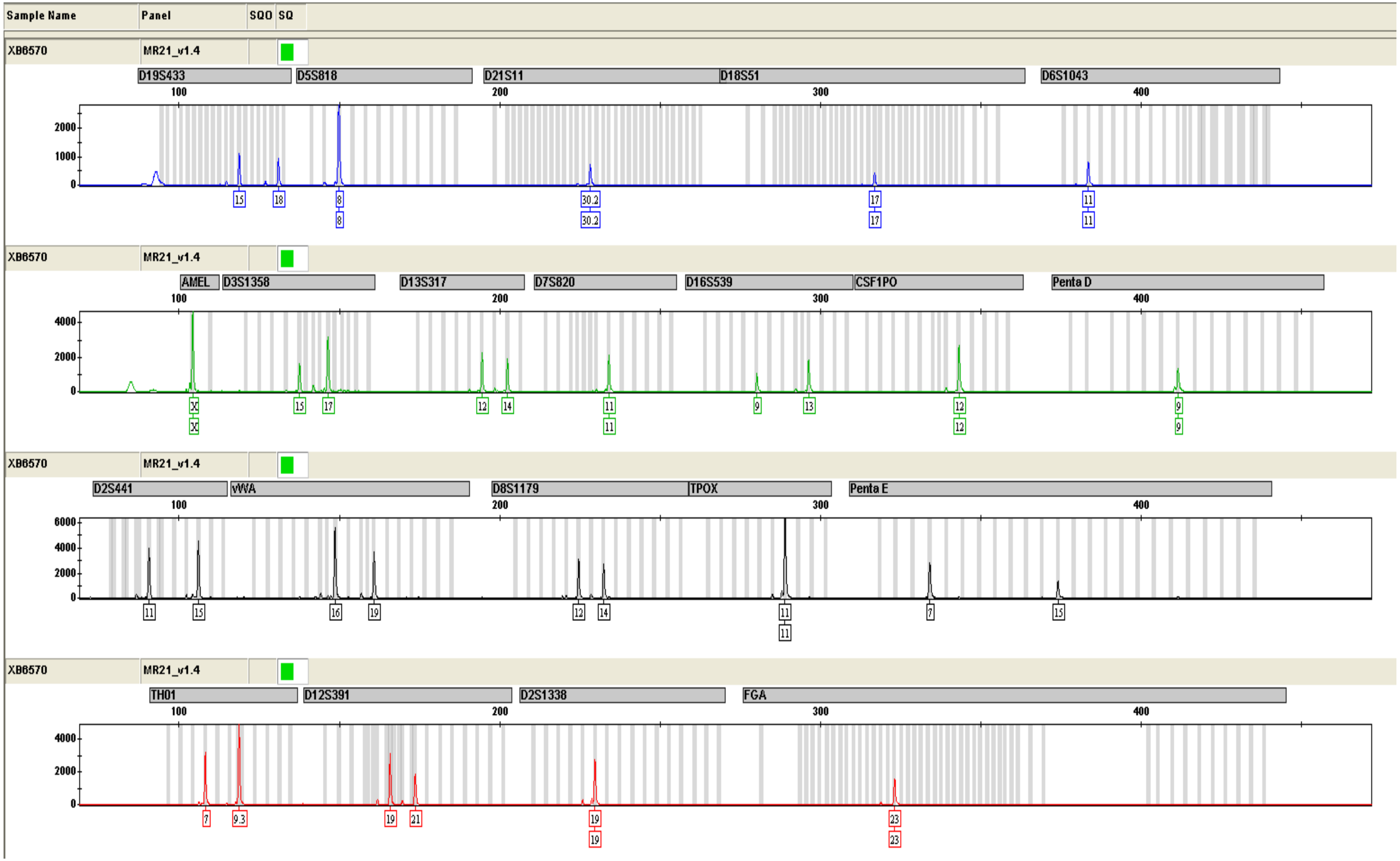

细胞遗传质量鉴定检测

Cell Line Authentication Service

STR基因型检测报告

样 品 信 息

样品编号：

客户样本编号	公司编号
MDA-MB-175	20191107-01

样品数量： 1

样品性状： 细胞系

检测项目： STR

检测方法： 用 Axygen 的基因组抽提试剂盒提取 DNA，采用 21- STR 扩增方案扩增，在 ABI 3730XL 型遗传分析仪上对 STR 位点和性别基因 Amelogenin 进行检测。

检 测 结 果

(一) 检验基本情况

公司编号	多等位基因	匹配细胞系	细胞库	EV 值	匹配说明
20191107-01	无	MDA-MB-175-VII	ATCC	1.0	完全匹配

样本基因型检验结果

- 多等位基因指三等位及以上基因现象。
- 本次检测各细胞分型结果良好。

(二) 各样本描述

- 20191107-01：该株细胞 DNA 分型在细胞系检索中找到**完全匹配**的细胞系，ATCC 数据库显示细胞名为 **MDA-MB-175-VII**，细胞号对应 **HTB-25**。本次检测在该细胞系中**没有发现多等位基因**。

备注： 待测细胞系与收录于 ATCC, DSMZ, JCRB 和 RIKEN 数据库的细胞系 STR 数据进行比对，未收录于以上细胞库的细胞系将无法匹配。

(三) 样本分型结果

细胞的 STR 位点和 Amelogenin 位点的基因分型结果

Loci	送检细胞 STR 信息			细胞库细胞 STR 信息		
	送检细胞名：MDA-MB-175			细胞库细胞名：MDA-MB-175-VII		
	Allele1	Allele2	Allele3	Allele1	Allele2	Allele3
D5S818	11	11		11	11	
D13S317	11	12		11	12	
D7S820	11	12		11	12	
D16S539	11	13		11	13	
VWA	15	16		15	16	
TH01	7	7		7	7	
AMEL	X	X		X	X	
TPOX	6	8		6	8	
CSF1PO	7	8		7	8	
D12S391	18	20				
FGA	23	26				
D2S1338	17	22				
D21S11	29	30				
D18S51	15	15				
D8S1179	14	17				
D3S1358	16	18				
D6S1043	12	19				
PENTAE	8	15				
D19S433	12.2	15				
PENTAD	3.2	11				
D1S1656	17	17				

Sample File	Sample Name	Panel	OS	SQ
02_B01_CellLineAuthentication-1-1108.fsa	MDA-MB-175	21Plex_STR_Panel_v1.1		

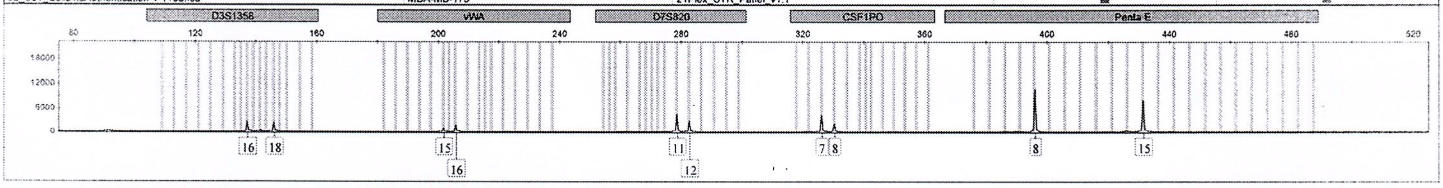

02_B01_CellLineAuthentication-1-1108.fsa	MDA-MB-175	21Plex_STR_Panel_v1.1		

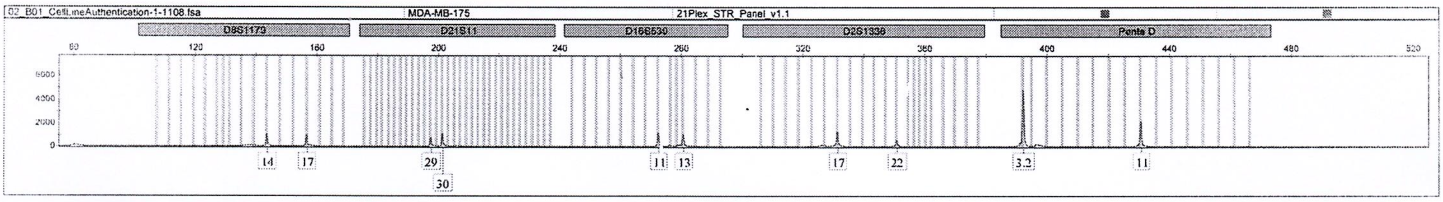

02_B01_CellLineAuthentication-1-1108.fsa	MDA-MB-175	21Plex_STR_Panel_v1.1		

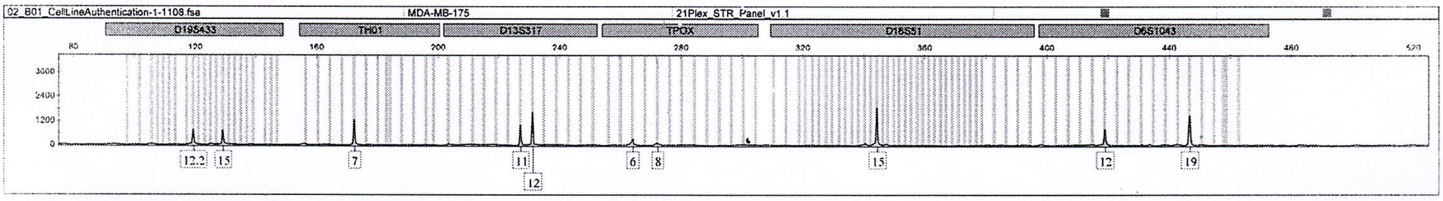

02_B01_CellLineAuthentication-1-1108.fsa	MDA-MB-175	21Plex_STR_Panel_v1.1		

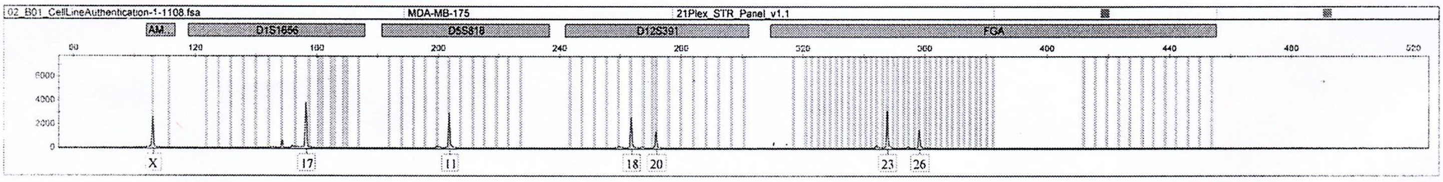

ZR-75-1 细胞 STR 鉴定报告

一、 材料处理和检验方法

取适量 **ZR-75-1** 细胞(1×10^6)使用 PureLink® Genomic DNA Mini Kit（美国 Life K182001)提前基因组 DNA，采用 PowerPlex®18D 系统(美国 Promega DC1802）试剂盒进行扩增，在 ABI3500 Genetic Analyzer（美国 Life3500）进行检测。

二、 检测结果

实验中阴性及阳性对照结果均正确。

ZR-75-1 细胞株的 STR 位点和 Amelogenin 位点的基因分型结果见附表，分型图谱见附图。

三、 分析说明

ZR-75-1 细胞株基因组 DNA 扩增后图谱清晰，分型结果良好。

四、 检验结论

1. **ZR-75-1** 细胞株 DNA 进行细胞 STR 分型结果显示，细胞株中未发现人类细胞交叉污染。
2. 该细胞株 DNA 分型在 ATCC 细胞库中找到与其细胞分型 100%相匹配的细胞株，细胞株名称为 **ZR-75-1**。

附表 1：细胞株 ZR-75-1 的 STR 位点和 Amelogenin 位点的基因分型结果

细胞 **ZR-75-1**（图片编号为 PC91）		
Marker	***Allele 1***	***Allele 2***
D3S1358	15	16
**THO1**	7	9.3
D21S11	31	31
D18S51	13	14
Penta E	7	14
**D5S818**	13	13
**D13S317**	9	9
**D7S820**	10	11
**D16S539**	11	11
**CSF1PO**	10	11
Penta D	14	14
**AMEL**	X	X
**vWA**	16	18
D8S1179	11	13
**TPOX**	8	8
FGA	20	22
D6S1043	12	12
D2S1338	16	25
D12S391	21	21
D19S433	13	14
D1S1656	16.3	16.3

附图 1：ATCC 官网 ZR-75-1 细胞 STR 位点信息

ZR-75-1 (ATCC® CRL-1500™)

Organism: ***Homo sapiens*, human** / Cell Type: **epithelial** / Tissue: **mammary gland**
Disease: **ductal carcinoma**

GENERAL INFORMATION | CHARACTERISTICS | CULTURE METHOD | SPECIFICATIONS

STR Profile

Amelogenin: X
CSF1PO: 10,11
D13S317: 9
D16S539: 11
D5S818: 13
D7S820: 10,11
THO1: 7,9.3
TPOX: 8
vWA: 16,18

附图 2：ZR-75-1 细胞 STR 位点和 Amelogenin 位点的基因分型结果

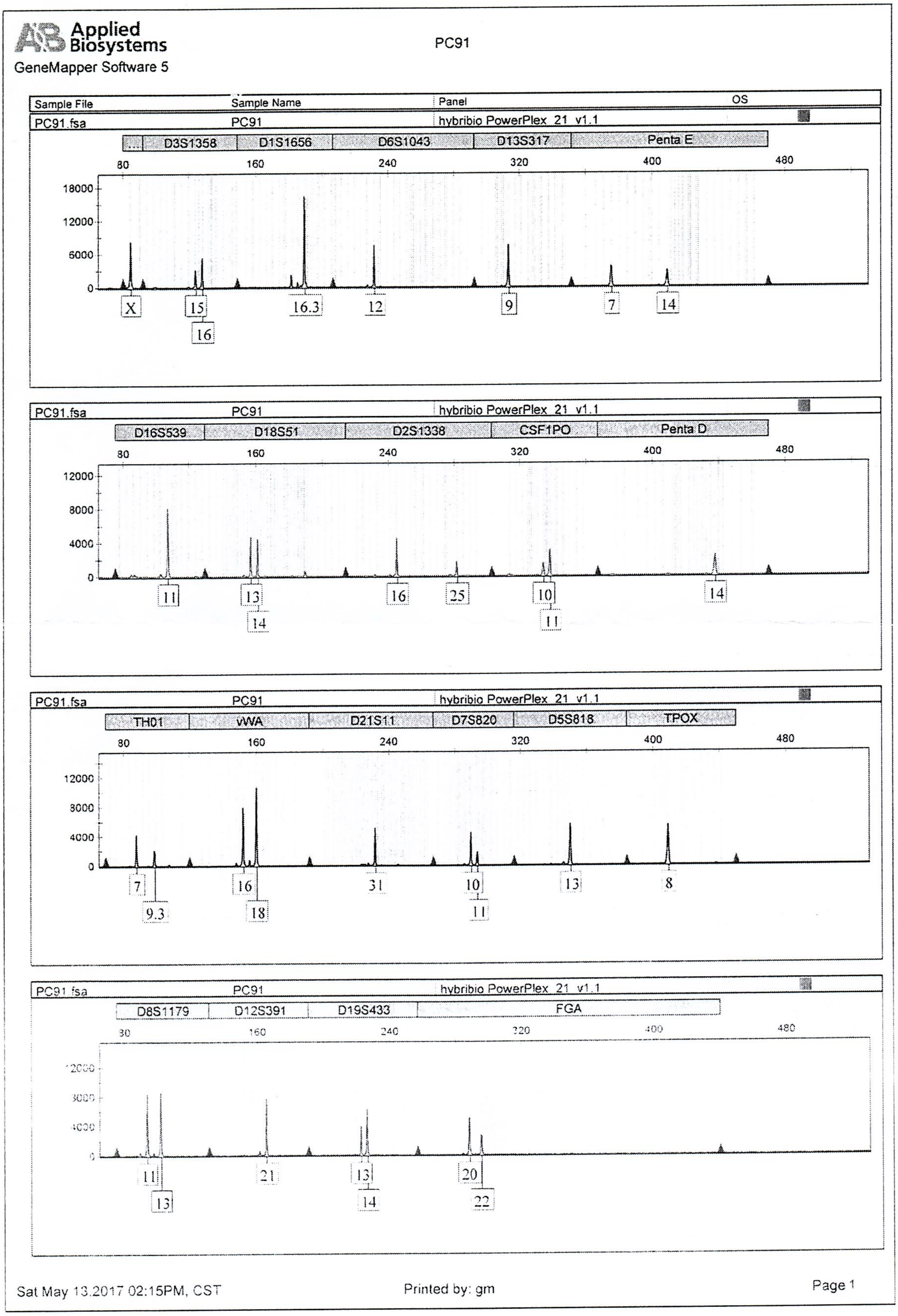

网站：www.procell.com.cn
电话：400-650-3656
邮箱：sales@procell.com.cn
