# Supplementary material for: Regulation of P53 signaling in breast cancer by the E3 ubiquitin ligase RNF187
Source: Cell Death Dis. 2022 Feb 14;13(2):149. doi: 10.1038/s41419-022-04604-3 (PMC8844070; doi:10.1038/s41419-022-04604-3)
Supplement: Supplementary file 9 — Authorship change form [file 41419_2022_4604_MOESM9_ESM.pdf]

SPRINGER NATURE

Change of authorship request form - Journals (pre-acceptance)

Important information. Please read.

- This form should be used by authors to request any change in authorship (adding/deleting authors) including changes in corresponding authors. This form should not be used for name changes. Please fully complete all sections. Use black ink and block capitals and provide each author's full name with the given name first followed by the family name.

- By signing this declaration, all authors guarantee that the order of the authors are in accordance with their scientific contribution, if applicable as different conventions apply per discipline, and that only authors have been added who made a meaningful contribution to the work.

- Please note, in author collaborations where there is formal agreement for representing the collaboration, it is sufficient for the representative or legal guarantor (usually the corresponding author) to complete and sign the Authorship Change Form on behalf of all authors, **next to the added/removed author(s)**. **(Complete Section 3, followed by Section 6.)**
  In author collaborations where there is no formal agreement for representing the collaboration and **there are more than 10 authors**, one may sign for all, provided the signer appends correspondence that attests that each of the authors have agreed to the change **and the added/removed authors sign the form**. **(Complete Section 3, followed by Section 6.)**

- Please note, we cannot investigate or mediate any authorship disputes. If you are unable to obtain agreement from all authors (including those who you wish to be removed) you must refer the matter to your institution(s) for investigation. Please inform us if you need to do this.

- If you are not able to return a fully completed form within **30 days** of the date that it was sent to the author requesting the change, we may have to withdraw your manuscript. We cannot publish manuscripts where authorship has not been agreed by all authors (including those who have been removed).

- Incomplete forms will be rejected.

- Please return/upload this form, fully completed, to the Journals Editorial Office. The Journal and/or Publisher will consider the information you have provided to decide whether to approve the proposed change in authorship. We may decide to contact your institution for more information or undertake a further investigation, if appropriate, before making a final decision.

SPRINGER NATURE Change of authorship request form - Journals (pre-acceptance)

Section 1: Please provide the current title of manuscript

Manuscript ID no.: CDDIS-18-3436

Title: Regulation of P53 signaling in breast cancer by the E3 ubiquitin ligase RNF187

Section 2: Please provide the previous authorship, in the order shown on the manuscript before the changes were introduced. Please indicate the corresponding author by adding (CA) behind the name.

	First name(s)	Family name	ORCID or SCOPUS id, if available
1st author	XIN	LI	
2nd author	ZHIGUO	NIU	
3rd author	HUIJIE	YANG	
4th author	XIAO	YANG	
5th author	YUN	LIU	
6th author	CHENG	YAN	
7th author	CHEN	SUN	
8th author	ZHONGBO	LI	
9th author	QI	CAO	
10th author	YINLU(CA)	DING	

Please use an additional sheet if there are more than 10 authors.

SPRINGER NATURE Springer Nature is one of the world's leading global research, educational and professional publishers, created in May 2015 through the combination of Nature Publishing Group, Palgrave Macmillan, Macmillan Education and Springer Science+Business Media.

SPRINGER NATURE

Change of authorship request form - Journals (pre-acceptance)

Section 1: Please provide the current title of manuscript

Manuscript ID no.: CDDIS-18-3436

Title: Regulation of p53 signaling in breast cancer by the E3 ubiquitin ligase RNF187

Section 2: Please provide the previous authorship, in the order shown on the manuscript before the changes were introduced. Please indicate the corresponding author by adding (CA) behind the name.

	First name(s)	Family name	ORCID or SCOPUS id, if available
11th author	TING(CA)	ZHUANG	0000-0002-6308-1253
12th author	JIAN(CA)	ZHU	0000-0003-3596-4339
13th author			
14th author			
15th author			
16th author			
17th author			
18th author			
19th author			
20th author			

Please use an additional sheet if there are more than 10 authors.

SPRINGER NATURE Springer Nature is one of the world's leading global research, educational and professional publishers, created in May 2015 through the combination of Nature Publishing Group, Palgrave Macmillan, Macmillan Education and Springer Science+Business Media.

Change of authorship request form - Journals (pre-acceptance)

SPRINGER NATURE

Section 3: Please provide a justification for change. Please use this section to explain your reasons for changing the authorship of your manuscript, e.g. what necessitated the change in authorship? Please refer to the (journal) policy pages for more information about authorship. Please explain why omitted authors were not originally included and/or why authors were removed on the submitted manuscript.

Since the reviews asked us to revise the experiments, Dr. Zhuo carried out the Western blot and the FACS. Dr. Zhuo also performed the statistical analysis. Dr. Sun Dr. Sun did the immunofluorescence. Dr. Ji performed the bioinformatics analysis. So we added Dr. Zhuo as a co-first author and changed Dr. Sun into a co-first author, and added Dr. Ji as an author.

Section 4: Proposed new authorship. Please provide your new authorship list in the order you would like it to appear on the manuscript. Please indicate the corresponding author by adding (CA) behind the name. If the Corresponding Author has changed, please indicate the reason under section 3.

	First name(s)	Family name (this name will appear in full on the final publication and will be searchable in various abstract and indexing databases)	Affiliated institute	E-mail address
1st author	XIN	LI	Xinxiang Medical University	lixin12036@163.com
2nd author	ZHIGUO	NIU	Xinxiang Medical University	niuzhiguo@xxmu.edu.cn
3rd author	SHU	ZHUO	Harvard School of Dental Medicine	zhuoshu1110@gmail.com
4th author	CHEN	SUN	The First Affiliated Hospital of Zhengzhou University	598129065@qq.com
5th author	HUIJIE	YANG	Xinxiang Medical University	1205283658@qq.com
6th author	XIAO	YANG	Xinxiang Medical University	775013101@qq.com
7th author	YUN	LIU	Xinxiang Medical University	1425075258@qq.com
8th author	CHENG	YAN	Xinxiang University	yanchengxx@163.com
9th author	ZHONGBO	LI	Xinxiang Medical University	735396587@qq.com
10th author	QI	CAO	Xinxiang Medical University	qi.cao@xxmu.edu.cn

Please use an additional sheet if there are more than 10 authors.

SPRINGER NATURE Springer Nature is one of the world's leading global research, educational and professional publishers, created in May 2015 through the combination of Nature Publishing Group, Palgrave Macmillan, Macmillan Education and Springer Science+Business Media.

SPRINGER NATURE

Change of authorship request form - Journals (pre-acceptance)

Section 3: Please provide a justification for change. Please use this section to explain your reasons for changing the authorship of your manuscript, e.g. what necessitated the change in authorship? Please refer to the (journal) policy pages for more information about authorship. Please explain why omitted authors were not originally included and/or why authors were removed on the submitted manuscript.

Section 4: Proposed new authorship. Please provide your new authorship list in the order you would like it to appear on the manuscript. Please indicate the corresponding author by adding (CA) behind the name. <u>If the Corresponding Author has changed, please indicate the reason under section 3</u>.

	First name(s)	Family name (this name will appear in full on the final publication and will be searchable in various abstract and indexing databases)	Affiliated institute	E-mail address
11th author	GUIMEI	JI	The Second Hospital, Shandong University	17620027119@163.com
12th author	YINLU(CA)	DING	The Second Hospital, Shandong University	dingyinlu@126.com
13th author	TING(CA)	ZHUANG	Xinxiang Medical University	77090993@qq.com
14th author	JIAN(CA)	ZHU	The Second Hospital, Shandong University	jian.zhu@email.sdu.edu.cn
15th author				
16th author				
17th author				
18th author				
19th author				
20th author				

Please use an additional sheet if there are more than 10 authors.

SPRINGER NATURE Springer Nature is one of the world's leading global research, educational and professional publishers, created in May 2015 through the combination of Nature Publishing Group, Palgrave Macmillan, Macmillan Education and Springer Science+Business Media.

Change of authorship request form - Journals (pre-acceptance)

SPRINGER NATURE

Section 5: Author contribution, Acknowledgement and Disclosures. Please use this section to provide a new disclosure statement and, if appropriate, acknowledge any contributors who have been removed as authors and ensure you state what contribution any new authors made (if applicable per the journal or book (series) policy). Please ensure these are updated in your manuscript - <u>after approval of the change(s)</u> - as our production department will not transfer the information in this form to your manuscript.

New acknowledgements:

no change to existing statement

New Disclosures (financial and non-financial interests, funding):

no change to existing statement

New Author Contributions statement (if applicable per the journal policy):

JZ, TZ and YL D conceived the designed that study. XL, ZG N and SZ performed the molecular and cellular biology of the study. XL, ZG N and CS performed the cellular phenotype assays. HJ Y, XY and YL performed the xenograft mice study. CY, CS, GM J, ZB L performed the RNA-sequence data analysis and bioinformatics data analysis. JZ and QC wrote the manuscript and approved the manuscript. YL D, TZ and JZ offered the funding support and project supervision during the revision stage.

State 'Not applicable' if there are no new authors.

SPRINGER NATURE Springer Nature is one of the world's leading global research, educational and professional publishers, created in May 2015 through the combination of Nature Publishing Group, Palgrave Macmillan, Macmillan Education and Springer Science+Business Media.

SPRINGER NATURE

Change of authorship request form - Journals (pre-acceptance)

Section 6: Declaration of agreement. *All* authors, unchanged, new and removed *must* sign this declaration.
(NB: Please print the form, (docu)-sign and return/upload a scanned copy. Please note that signatures that have been inserted as an image file are acceptable as long as it is handwritten. Typed names in the signature box are unacceptable.) *** Please delete as appropriate. Delete all of the bold if you were on the original authorship list and are remaining as an author.**

	First name	Family name		Signature	Date
1st author	XIN	LI	I agree to the proposed new authorship shown in section 4	Xin Li	7/12/2021
2nd author	ZHIGUO	NIU	I agree to the proposed new authorship shown in section 4	Zhi Guo Niu	7/12/2021
3rd author	SHU	ZHUO	I agree to the proposed new authorship shown in section 4 **and the addition of my name to the authorship list**	Shu Zhuo	7/12/2021
4th authors	CHEN	SUN	I agree to the proposed new authorship shown in section 4	Chen Sun	7/12/2021
5th author	HUIJIE	YANG	I agree to the proposed new authorship shown in section 4	Huijie Yang	7/12/2021
6th author	XIAO	YANG	I agree to the proposed new authorship shown in section 4	Xiao Yang	7/12/2021
7th author	YUN	LIU	I agree to the proposed new authorship shown in section 4	Yun Liu	7/12/2021

SPRINGER NATURE Springer Nature is one of the world's leading global research, educational and professional publishers, created in May 2015 through the combination of Nature Publishing Group, Palgrave Macmillan, Macmillan Education and Springer Science+Business Media.

Change of authorship request form - Journals (pre-acceptance)

SPRINGER NATURE

Section 6: Declaration of agreement. *All* authors, unchanged, new and removed *must* sign this declaration.
(NB: Please print the form, (docu)-sign and return/upload a scanned copy. Please note that signatures that have been inserted as an image file are acceptable as long as it is handwritten. Typed names in the signature box are unacceptable.) *** Please delete as appropriate. Delete all of the bold if you were on the original authorship list and are remaining as an author.**

	First name	Family name		Signature	Date
8th author	CHENG	YAN	I agree to the proposed new authorship shown in section 4	Cheng Yan	7/12/2021
9th author	ZHONGBO	LI	I agree to the proposed new authorship shown in section 4	Zhongbo Li	7/12/2021
10th author	QI	CAO	I agree to the proposed new authorship shown in section 4	Qi Cao	7/12/2021
11th authors	GUIMEI	JI	I agree to the proposed new authorship shown in section 4 **and the addition of my name to the authorship list**	Guimei Ji	7/12/2021
12th author	YINLU	DING	I agree to the proposed new authorship shown in section 4	Yinlu Ding	7/12/2021
13th author	TING	ZHUANG	I agree to the proposed new authorship shown in section 4	Ting Zhuang	7/12/2021
14th author	JIAN	ZHU	I agree to the proposed new authorship shown in section 4	Jian Zhu	7/12/2021

SPRINGER NATURE Springer Nature is one of the world's leading global research, educational and professional publishers, created in May 2015 through the combination of Nature Publishing Group, Palgrave Macmillan, Macmillan Education and Springer Science+Business Media.

SPRINGER NATURE

Change of authorship request form - Journals (pre-acceptance)

	First name	Family name		Signature	Date
8th author			I agree to the proposed new authorship shown in section 4 **/and the addition/removal*of my name to the authorship list** /and the proposed change in corresponding author		
9th author			I agree to the proposed new authorship shown in section 4 **/and the addition/removal*of my name to the authorship list** /and the proposed change in corresponding author		
10th author			I agree to the proposed new authorship shown in section 4 **/and the addition/removal*of my name to the authorship list** /and the proposed change in corresponding author		

Please use an additional sheet if there are more than 10 authors.

In case of author collaborations with formal agreement:

	Name of consortium/consortia	First name	Family name		Signature	Date
Representative/legal guarantor	The Second Hospital, Shandong University	JIAN	ZHU	I agree to the proposed new authorship shown in section 4	Jian Zhu	7/12/2021

Both added/removed authors should complete the information in the first table under Section 6.

---- End of form ----

SPRINGER NATURE Springer Nature is one of the world's leading global research, educational and professional publishers, created in May 2015 through the combination of Nature Publishing Group, Palgrave Macmillan, Macmillan Education and Springer Science+Business Media.
